# Pangenomic structural variant patterns reflect evolutionary diversification in *Brassica napus*

**DOI:** 10.1186/s13059-025-03833-x

**Published:** 2025-11-10

**Authors:** Nazanin P. Afsharyan, Agnieszka A. Golicz, Rod J. Snowdon

**Affiliations:** 1 Department of Plant Breeding, Justus Liebig University Giessen, Giessen, 35392 Germany; 2 Leibniz Centre for Agricultural Landscape Research (ZALF), Müncheberg, 15374 Germany; 3 Department of Agrobioinformatics, Justus Liebig University Giessen, Giessen, 35392 Germany

**Keywords:** Structural variation, Intraspecific diversification, Rapeseed, Oilseed rape, Swede

## Abstract

**Background:**

Understanding genetic diversity is crucial for enhancing crop productivity. This study explores species-wide genome structural variation (SV) and its role in intraspecific and ecogeographical diversification of *Brassica napus*, a recently evolved, globally important allopolyploid crop.

**Results:**

We perform whole-genome long-read DNA-sequencing and construct reference-guided genome assemblies for 94 accessions, including winter-type, spring-type, and East Asian oilseed, along with kale forms and swedes/rutabagas. We investigate pangenomic patterns of SVs and determine pangenome-wide distributions and frequencies of inversions, gene presence-absence variants, and collective SVs including insertions and deletions. Results reveal pangenome-wide patterns for insertions, deletions, inversions, and large chromosomal deletions/duplications, reflecting evolutionary diversification across morphotypes and ecotypes. Collective SVs are unevenly distributed and biased toward subgenome A, with asymmetrical selection pattern favoring subgenome C. Selection signatures for inversions exhibit no subgenome asymmetry; however, increased selection signal strength and frequency are detected in paracentric chromosome regions, highlighting evolutionary significance. Analysis of the candidate loci under selection identifies regions for collective SVs and inversions, harboring genes for organ formation, cell division and expansion in swede, and stress responses in East Asian oilseed rape. Large chromosomal duplications and deletions distinguish swede from oilseed rape, particularly in subgenome C, including copy-number variation in flowering-time genes *BnFLC.C09* and *BnATX2.C08*, and cell wall development gene *BnCEL2.C08*.

**Conclusions:**

These findings underscore functional and evolutionary significance of pangenomic SV formation during *Brassica napus* diversification. Information on SV patterns with putative functional relevance provides breeding insights, particularly for developing molecular markers to optimize performance of *Brassica napus* and other Brassica crops.

**Supplementary Information:**

The online version contains supplementary material available at 10.1186/s13059-025-03833-x.

## Background

Genomic structural variants (SVs) are abundant chromosomal or genic variations such as deletions, insertions, duplications, inversions, translocations, chromosomal losses and gains, and complex DNA rearrangements [1, 2]. SVs are typically defined as ≥ 50 base pairs (bp) in size [2], to distinguish them from smaller variants like single-nucleotide polymorphisms (SNPs) and small insertions or deletions (indels), though thresholds as low as 30 bp are sometimes used to avoid technical limitations in SV detection [3]. Such variants lead to segmental gene presence-absence variation (PAV) and copy-number variation (CNV). In polyploids with multiple similar subgenomes, chromosome-scale homoeologous exchanges are also frequent [4–6]. All of these events can impact genes, and hence, influence all kinds of agronomic traits in crops [7]. SV events have been shown to play a crucial role in adaptation and domestication of crops, as they affect gene structure and genomic regulatory regions more significantly than smaller variants such as SNPs. SVs form through various mechanisms, including double-strand break repair (e.g., non-homologous end joining, microhomology-mediated end joining), replication errors, recombination errors, or meiotic crossovers among non-homologous chromosomes [1]. Among these, transposable elements (TEs) are major contributors to SV formation in the human genome. Beyond their mobility via retrotransposition, TEs also provide homologous sequences that act as substrates for ectopic recombination. These TE-mediated rearrangements (TEMRs) can result in deletions, duplications, inversions, and complex structural alterations [8].

Polyploidy in plants can result from spontaneous genome duplication (autopolyploidy) or interspecific hybridization (allopolyploidy). Many recent polyploid crops have undergone multiple rounds of polyploidization [9] or have been subjected to introgressive hybridization with gene pools of different ploidy levels [10, 11], enabling them to adapt to new environments [12]. Plant genomes, characterized by high levels of repetitive sequences and varying degrees of polyploidy, add complexity to the relationship between SVs and the occurrence of crossovers during meiosis. For example, recent studies have shown that large SVs, particularly inversions, can suppress recombination. However, crossovers within these SVs can sometimes occur, and depending on their relative position to the centromere, these may result in unbalanced gametes with dicentric and/or acentric chromosomes, or balanced gametes with new duplications and deletions [13].

The major crop species *Brassica napus* L. (genome AACC, 2n = 38) is estimated to have emerged no later than around 7000 years ago [4, 14, 15] from the spontaneous hybridization of its diploid progenitors *B. rapa* and *B. oleracea*. Because no wild *B. napus* forms are known, the actual hybridization event likely did not occur before the early middle ages, when the two progenitor species from Asia and the Mediterranean region are thought to have been cultivated together for the first time as a result of human trade and exchange along the Silk Road. Subspecies *B. napus* ssp. *napus* is today a globally important oilseed crop (rapeseed, oilseed rape, canola) which also includes leafy kale or fodder forms, while the other subspecies *B. napus* ssp. *napobrassica*, known as swede or rutabaga, has an enlarged hypocotyl that is consumed as a vegetable or used for livestock grazing [16]. Despite a short domestication history, *B. napus* exhibits remarkable phenotypic diversity and phenological adaptation. *B. napus* has adapted to diverse climate zones in major production areas across Europe, Asia, North and South America, and Australia, requiring broad diversity in adaptation traits including growth habit, vernalization requirement, winter hardiness, and phenology, from early-flowering spring types to late-flowering winter and biennial types. There are three major oilseed *B. napus* ecotypes within ssp. *napus*. Winter-type oilseed rape (WOSR) originated in Europe and is sown in Autumn, being adapted to cold winters with a stringent vernalization requirement and a longer life cycle [15]. Spring-sown oilseed rape (SOSR), known particularly in North America as canola, was domesticated in Europe around 1700 and has no vernalization requirement [17]. An intermediate form, semi-winter oilseed rape, was developed in East Asia over the past 200 years for adaptation to milder winters [18]. In breeding efforts to improve local adaptation and disease resistance of semi-winter oilseed rape, interspecific crosses with diploid progenitors, particularly the subgenome A donor *B. rapa*, have been used [19]. Swede (ssp. *napobrassica*) represents a morphologically and genetically distinct group selected primarily for root development and biomass traits as it is distinguished by an enlarged hypocotyl. The origin of *B. napus*, including swede, remains debated with recent studies suggesting a single hybridization event [20], while others suggest a few independent hybridization events [21]. Swede is thought to have been domesticated before oilseed rape (OSR) in Europe, and gene introgression from *B. rapa* into swede [20] has been used particularly to transfer resistance to soil pathogens [22]. Major breeding breakthroughs in the 1960 s and 1970 s eliminated undesirable erucic acid in the oil and reduced aliphatic glucosinolates in the seed meal, drastically improving the value of oilseed forms as a high-quality source of vegetable oil and protein-rich livestock feed. Since then, intensive breeding in OSR has considerably improved oil content, seed yield, and disease resistance. However, these breeding efforts have also narrowed the gene pool of elite cultivars, particularly because only a few trait donors were utilized to establish the key seed quality traits [23].

*B. napus* is known as a valuable model for studying intraspecific diversification and ecogeographic adaptation. Recent genomic studies have revealed extensive chromosome-scale SV events contributing to this diversity. However, these studies also highlight the challenges of detecting whole-genome SVs in the allotetraploid genome of *B. napus* due to high homoeology between chromosomes and frequent homoeologous exchanges [4]. These challenges have been mitigated by recent advances in long-read sequencing technologies and bioinformatics tools, which allow for high-precision SV detection based on long-read alignments [24, 25] or accurate whole-genome assemblies [26].

Previous studies have investigated species-wide genetic variation, including SNPs [4, 15, 20, 27, 28], simple sequence repeats (SSRs) [29], copy number variations (CNVs) [30], and homoeologous chromosomal exchanges [31] in *B. napus* to study its origins and ecogeographical adaptation. Several of these studies used the global *B. napus* ERANET-ASSYST collection [29] to investigate genome-wide genetic variation using SSR, CNV, and SNP variants [29, 30, 32]. Recently, there has been growing interest in constructing pangenome references to describe the available genetic diversity within crop species. While pangenomes have historically transformed our understanding of microbial diversity [33], their application in plant genomics has led to understanding of plant genomic diversity and its impact on phenotypic variation. In plants, pangenome analyses have revealed extensive structural variation that is often absent from single-reference genomes and can underlie critical phenotypic traits. For instance, in *Arabidopsis thaliana*, pangenome studies identified SVs that overlapped with thousands of genes and revealed structural variants associated with ecotype-specific adaptations [34]. Similarly, in *Brassica oleracea*, pangenomic studies identified SVs responsible for morphotype diversification by contributing to altering gene expression and regulatory regions [35]. These findings underscore the game-changing potential of pangenome approaches for capturing diversity within species and uncovering functional diversity and evolutionary dynamics. This highlights the limitations of single-reference genomes, both in capturing the full spectrum of genetic variation, and in achieving comprehensive and accurate identification and representation of genetic variants within a species [33]. Given the complex polyploid genome structure of *Brassica napus* and the phenotypic and ecogeographical diversity among its morphotypes, a species-wide SV analysis within a comprehensive pangenome approach can provide crucial insights into underlying intraspecific diversification and inform breeding strategies aimed at crop improvement. Advances in high-throughput, cost-effective DNA sequencing technologies have led to a significant increase in the development of pangenome references for major crops, including rice (*Oryza sativa* L.) [36, 37], maize (*Zea mays* L.) [38], soybean (*Glycine max* L.) [39], and rapeseed [40, 41]. Recent publications have also introduced pangenome SV catalogs for *Arabidopsis* [42] and a pangenome inversion index for rice [43]. Despite the growing body of research on functional variation caused by SVs, and the increasing precision and affordability of third-generation sequencing technologies, no large-scale study has yet surveyed the species-wide SV landscape in *B. napus*, or its connection to intraspecific diversification.

In this study, we analyzed 94 homozygous, ecogeographically diverse accessions of *B. napus* from the ERANET-ASSYST diversity collection to explore the pangenomic SV landscape and its relationship to intraspecific diversification. Based on alignment to the gold-standard *B. napus* Darmor-bzh v10 reference genome [44], we described and characterized pangenome-wide distribution and frequencies for gene presence-absence variants, inversions, and collective SVs. We performed long-read DNA sequencing using Oxford Nanopore Technology PromethION R9.4.1 flow cells, achieving an average 39.48 × whole-genome coverage. This data was used to construct reference-guided genome assemblies. SV calling and inversion detection were performed using two approaches: long-read alignments and genome assembly alignments. We conducted genome-wide analyses of phylogeny, candidate loci under selection, and presence-absence variation between different ecotypes and morphotypes. Additionally, we investigated large chromosomal deletions and duplications and analyzed their occurrence in different morphotypes.

## Results

### Generating high-coverage genome-wide long-read DNA sequencing data

Using Oxford Nanopore technology, we performed long-read whole-genome DNA sequencing of 94 *B. napus* accessions (Additional file 1: Table S1) that span the range of genetic diversity represented by the ERANET-ASSYST diversity set [29]. Overall, we generated approximately 39 Tb of FAST5 data. Given the *B. napus* Darmor-bzh v10 (D10) reference genome size of 1132 Mbs [44], coverage ranged between 21.63 × and 71.52 × (24.49 Gb to 80.97 Gb of FASTQ data per accession), with an average coverage of 39.48 ×. N50 values ranged from 18.00 to 54.61 kbp, with an average of 29.37 kbp (Additional file 1: Table S2).

### Constructing alignment-based genome assemblies

We constructed genome assemblies for all 94 *B. napus* accessions by building de novo contigs using long reads, followed by alignment-based scaffolding based on the D10 reference assembly. The average assembly size was 0.89 Gb across 19 chromosomes, with individual assembly sizes ranging from 0.66 to 1.01 Gb. The assemblies were evaluated using Benchmarking Universal Single-Copy Orthologs (BUSCO), revealing that on average, 99.57% of 1614 complete BUSCO genes were detected across the 94 assemblies (Additional file 1: Table S3). Of these, the average percentage of complete single-copy and duplicated BUSCOs was 5.48 and 94.09%, respectively, reflecting the polyploid nature of *B. napus*.

### Determining frequency of collective SVs demonstrated pangenome-wide SV distribution in intergenic and intragenic regions

**Fig. 1:**
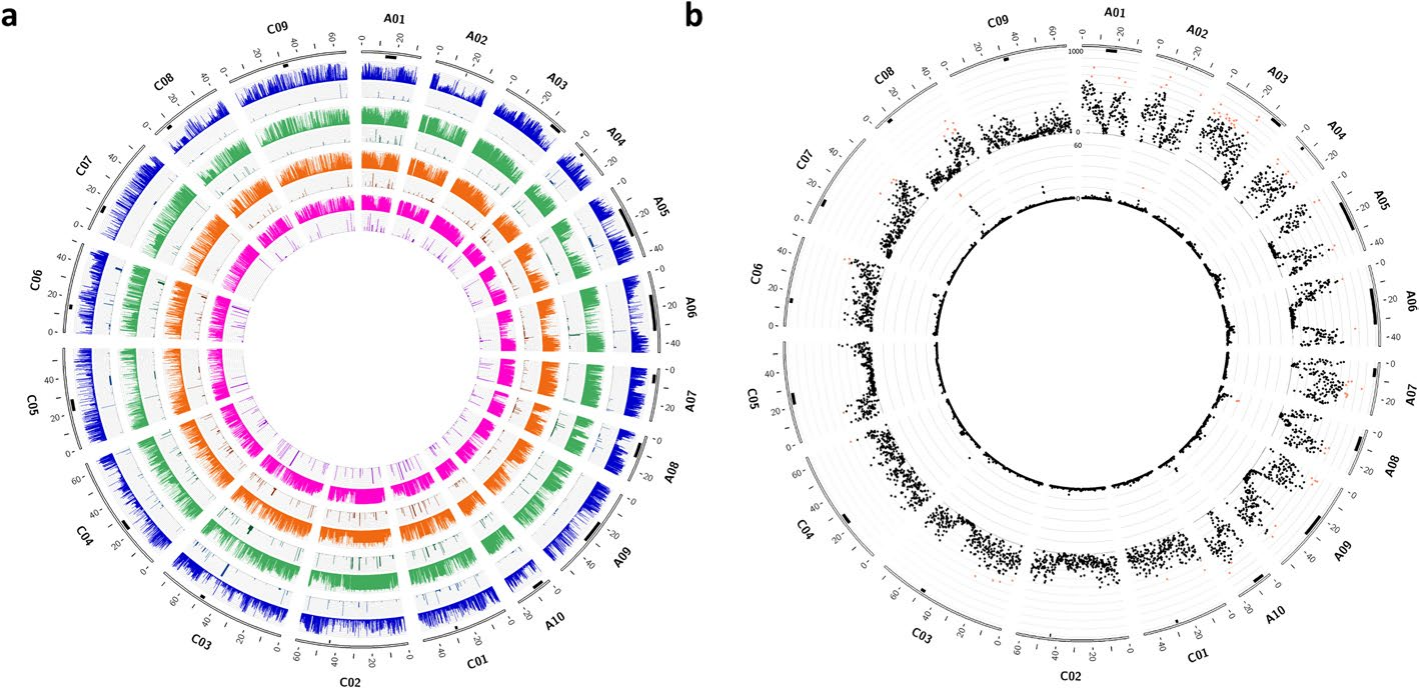
Overview of pangenome-wide distribution and frequency of collective SVs (Pan-CSV) and inversions (Pan-INV) in a panel of 94 homozygous, ecogeographically diverse accessions of *B. napus*. **a** The distribution (*x*-axis) and frequency (*y*-axis, 0 to 100) of SV events for the pan-CSV and pan-INV. For each ecotype group, the histogram on top describes pan-CSV, while the histogram on the bottom shows pan-INV. The circos plot from outer to inner layer includes chromosomes, centromeric regions (marked in black), WOSR (blue), SOSR (green), East Asian OSR (orange), and swede/rutabaga (magenta) accessions. The frequency is normalized as a percentage. **b** Chromosome distribution (*x*-axis) and number (*y*-axis, outer plot: 0 to 1000, inner plot: 0 to 60) of hotspots for pan-CSV (outer plot) and pan-INV (inner plot). The chromosomes are shown as the outer layer, followed by centromeric regions (marked in black). The dot plots display the number per 200 kbp window. The red dots indicate the top 30% per 200 kbp window, calculated separately for each subgenome. The coordinates of the centromeric regions were determined as detailed by Rousseau-Gueutin et al. [44]. Plots were generated using Circos [105]

SVs including insertions, deletions, inversions, and translocations (denoted here as collective SVs) were detected after aligning long reads to the D10 reference genome. The mapping efficiency of the filtered reads was 100%. Total 69,004 SVs from the call set overlapped with large deleted chromosome segments and were filtered-out as summarized in Additional file 1: Tables S4-5. The SV detection pipeline identified a grand total of 3,649,887 quality-filtered SVs across all accessions, consisting of 1,922,316 insertions, 1,727,416 deletions, 139 inversions, and 16 translocations (Fig. 1a, Additional file 1: Tables S6-7). In total, pangenome-wide frequencies of 861,307 SV events, up to 100 kbp in size, were determined based on long-read alignments including 755,613 insertions, 105,655 deletions, 23 inversions, and 16 translocations (Additional file 1: Table S8). Of 108,190 genes annotated in the D10 reference genome, 39.58% were found to contain SVs in their coding sequences (CDS) or untranslated regions (UTRs), including rare variants (Additional file 1: Table S9). Among these were 108 putative flowering-time-related genes (Additional file 1: Table S10), and 174 putative resistance (R) genes (Additional file 1: Table S11). Comparisons with previously reported SVs confirmed, for instance, a 288-bp SV in the *FLOWERING LOCUS T* gene on chromosome A02, as identified by Vollrath et al. [45] through both short-read sequencing and PCR validation. Additionally, we confirmed a 1.3-kbp SV in the putative promoter region of the same gene reported by Chawla et al. [46]. Furthermore, SVs in the *FLOWERING LOCUS C* gene on chromosome A10 included a 621-bp SV and a 4421-bp SV in the promoter region, and a 5565-bp SV in the first exon, all previously reported by Song et al. [40].

### Determining pangenome-wide distribution and frequency of inversions revealed link with intraspecific diversification

Reference-guided genome assemblies were aligned to the D10 reference genome to detect 1166 non-redundant inversion events, ranging in size from 199 bp to 12.097 Mbp, including 84 inversions larger than 1 Mbp (Fig. 1a, Additional file 1: Tables S12-13). These inversions were merged, and their pangenome-wide distribution and frequency was determined. Analysis of over-represented inversions in each ecotype group revealed 1, 8, and 15 inversions present in ≥ 70% of the accessions in the WOSR, SOSR, and swede/rutabaga groups, respectively. Among them a 506.4-kbp inversion was located on chromosome C05 which was present in all swede accessions but was not found in WOSR and SOSR ecotypes. On the other hand, there were 5 and 1 inversions that were under-represented (≤ 30%) in WOSR and SOSR respectively (Additional file 1: Table S14). The results showing the over-representation and under-representation of inversions across ecotype groups were further supported by statistical analysis using Fisher’s exact test, confirming the statistical significance of the associations (Additional file 1: Table S15).

### Hotspots for pangenome-wide collective SVs and inversions

We investigated SV hotspots in 200-kbp windows across all chromosomes for pangenome-wide distribution and frequencies of collective SVs and inversions. For subgenomes A and C, an average of 294.43 (SD = 189.23) and 131.67 (SD = 100.44) SV events per 200 kbp were detected for collective SVs, while for inversions, an average of 0.87 (SD = 1.99) and 0.44 (SD = 2.41) events were found for each subgenome, respectively. Statistical analysis showed significantly higher SV occurrence in subgenome A compared to subgenome C for both collective SVs (*p*-value = 1.68E*–*191) and inversions (*p*-value = 7.76E–11). Hotspots were defined as the top 30% of windows with the highest frequency of SVs. For the collective SVs, 55 hotspots were found in subgenome A and 23 in subgenome C, mostly toward telomeric regions (Fig. 1b, Additional file 1: Table S16). For the inversions, hotspots were found in chromosomes A02 and C08, near centromeres, and A08, within centromeric regions (Fig. 1b, Additional file 1: Table S17).

### Structural variants differentiate accessions by ecotype and subspecies

**Fig. 2:**
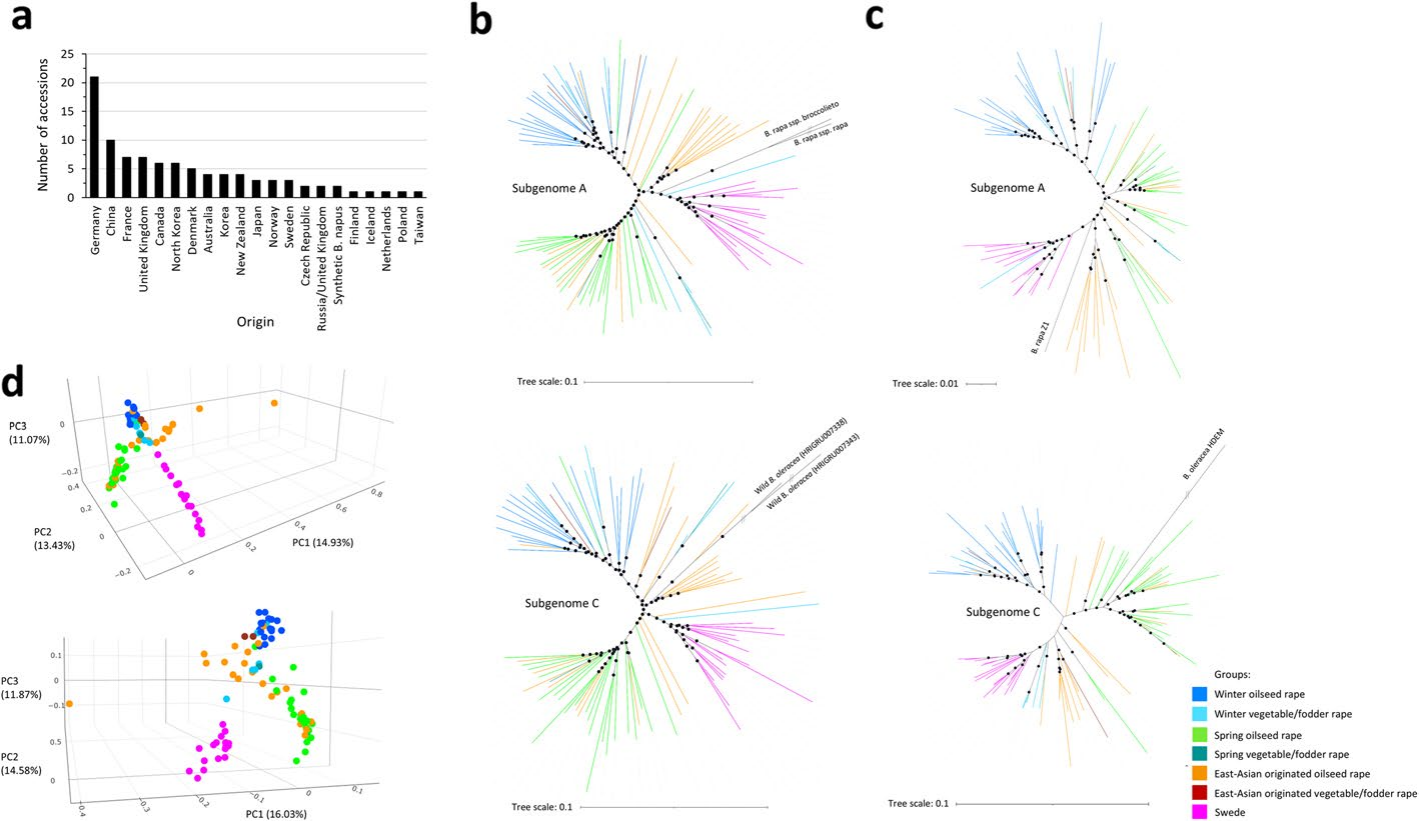
Phylogenetic analysis of 94 homozygous, ecogeographically diverse accessions of *B. napus* based on pangenome-wide collective SVs and inversions. **a** Overview of the origin and number of accessions. **b** Phylogenetic tree of A and C lineages for all accessions, constructed based on pangenome-wide distribution and frequency of collective SVs (pan-CSV). Two accessions of *B. rapa* (*B. rapa* ssp. *rapa* and *B. rapa* ssp. *broccolieto*) [71] and two accessions of wild *B. oleracea* (Genebank/cultivar ID: HRIGRU007343 and HRIGRU007338) [35] were included in the analysis for the A and C lineages, respectively. **c** Phylogenetic tree of A and C lineages for all accessions, constructed based on pangenome-wide distribution and frequency of inversions (pan-INV). *B. rapa* accession Z1 and *B. oleracea* accession HDEM [72], were included in the analysis for A and C lineages, respectively. Phylogenetic trees were constructed using a maximum likelihood approach. The reliability of the tree was also confirmed by 1000 bootstrap replicates, and clades with bootstrap values of above 50% are indicated by a black dot. Visualization was performed using the Interactive Tree Of Life online tool (https://itol.embl.de). **d** PCA plot of all accessions based on pan-CSV (top) and pan-INV (bottom)

Unrooted phylogenetic analyses for collective SVs were performed using 183,241 and 140,049 loci for subgenomes A and C, respectively; and for inversions, 684 and 577 inversion loci were used for subgenomes A and C, respectively. Unrooted phylogenetic analyses of the subgenomes A and C based on pangenome-wide collective SVs and inversions clustered most accessions into their respective WOSR, SOSR, and swede groups. As expected, East Asian OSR accessions were intermediate. The A lineage analysis grouped swedes and some East Asian OSR accessions closer to *B. rapa* for both collective SVs and inversions, while the C lineage analysis clustered some East Asian OSR accessions near wild *B. oleracea* for collective SVs (Fig. 2b) and grouped wild *B. oleracea* closer to SOSR and East Asian OSR accessions for inversions (Fig. 2c). Principal component analysis (PCA) supported these findings, clearly separating WOSR, SOSR, and swede groups for both collective SVs and inversions (Fig. 2d). Similarly, unrooted phylogenetic analyses of the subgenomes A and C based on SNP data clustered most accessions into their respective WOSR, SOSR, and swede groups, with East Asian OSR accessions appearing as intermediate. Both A and C lineage analyses grouped WOSR accessions close to progenitors (Additional file 2: Fig. S1).

### Characterization of the candidate loci under selection based on pangenome-wide collective SVs exhibited subgenome bias for intraspecific diversification

**Fig. 3:**
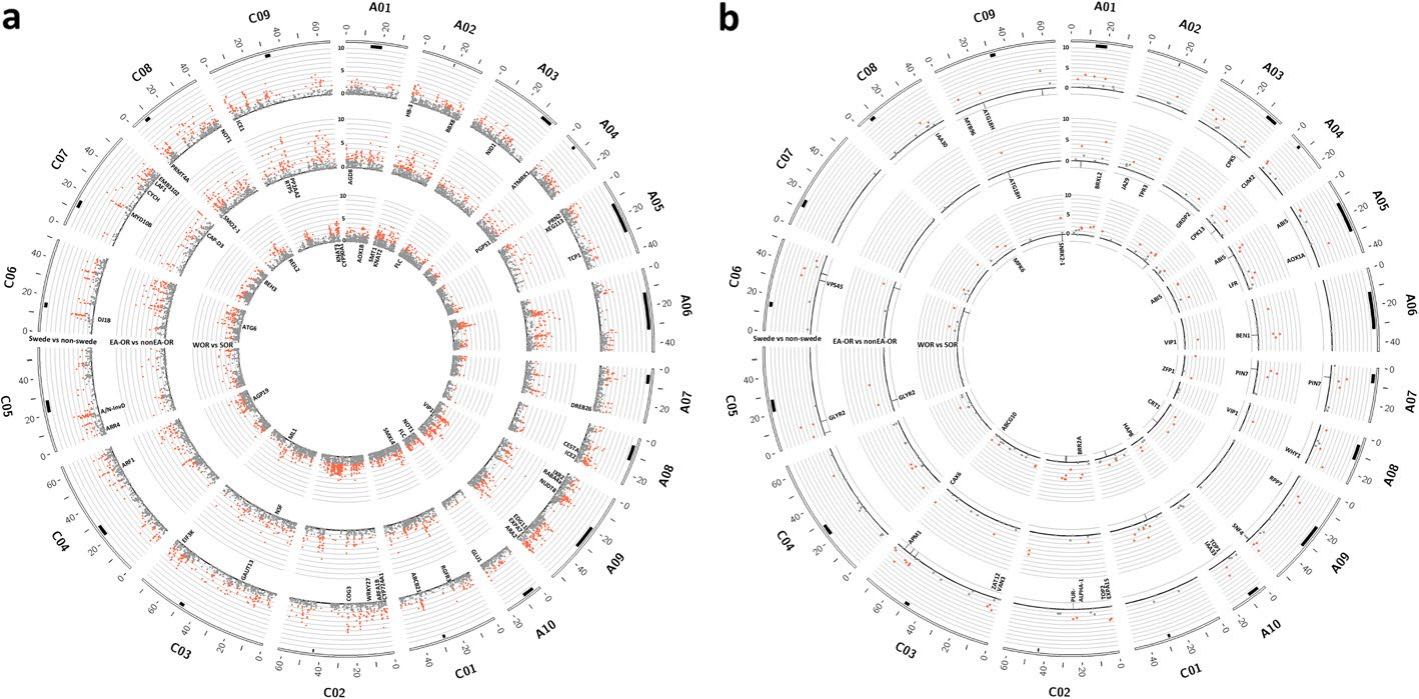
Overview of pangenome-wide screening for candidate loci under selection associated with intraspecific and ecogeographical diversification in *B. napus*. **a** Distribution of pangenome-wide *F*_ST_ values for collective SVs (pan-CSV). **b** Distribution of pangenome-wide *F*_ST_ values for inversions (pan-INV). Positions of the overlapping signals from collective SVs and inversions are marked below each Manhattan subplot in **b**. The chromosomes are shown as the outer layer, followed by centromeric regions (marked in black). The Manhattan plots, from outermost circle inwards, depict *F*_ST_ values for pairwise comparisons of swede vs. non-swede accessions, East Asian OSR vs. all other OSR accessions, and WOSR vs. SOSR accessions, respectively. A 50-kbp non-overlapping window was used to calculate *F*_ST_ values. *F*_ST_ values were normalized as *z*-scores. Dots colored in red indicate values above the significance level threshold of *α* = 0.05, corresponding to *z* = 1.645. A number of genes located within the regions under selection, known to be related to developmental and adaptation mechanisms, are presented under the respective Manhattan plot. The coordinates of the centromeric regions were determined as detailed by Rousseau-Gueutin et al. [44]. Plots were generated using Circos [105]

Using the pangenome-wide distribution and frequency of collective SVs, we investigated selection signatures contributing to diversification in *B. napus*, identifying key chromosome regions in pairwise comparisons of swede vs. non-swede, East Asian OSR vs. all other OSR, and WOSR vs. SOSR accessions (Fig. 3a; Additional file 1: Tables S18-20). Then, we determined the genes that were located in regions under selection (Additional file 1: Tables S21-23). Significantly higher average *z*-values for subgenome C were observed compared to A, for swede vs. non-swede (*p*-value = 5.05E–11) and WOSR vs. SOSR (*p*-value = 1.04E–09) pairwise comparisons. No significant differences in average *z*-values were noted for East Asian OSR vs. all other OSR accessions. GO enrichment analysis for swede vs. non-swede accessions revealed significant overrepresentation of genes involved in cell division and expansion, meiosis, and environmental responses and disease resistance (Additional file 1: Table S24). For East Asian OSR vs. all other OSR groups, regions under selection showed significant overrepresentation of genes involved in disease resistance, and abiotic stress response (Additional file 1: Table S25). Similarity, enriched GO terms in genomic regions under selection for WOSR vs. SOSR indicated presence of genes potentially involved in disease resistance and stress response (Additional file 1: Table S26). Additionally, the key flowering time genes, orthologs of Arabidopsis *FLOWERING LOCUS C* (*FLC*), in chromosomes A03 and A10 were also located near genomic regions that were identified to be putatively under selection for WOSR vs SOSR comparison. Since *FLC* is a key determinant of vernalization requirement, it can be expected that SVs within *B. napus* orthologs of this and other key adaptive genes might indeed have contributed to intraspecific selection in *B. napus*.

### Characterization of the candidate loci under selection based on pangenome-wide inversions highlighted stronger signals toward paracentric regions

The pangenome-wide distribution and frequency of inversions was used to explore selection signatures during domestication and breeding of *B. napus*. The results revealed chromosome regions which contribute to diversification in all pairwise comparisons, including swede vs. non-swede, East Asian OSR vs. all other OSR, and WOSR vs. SOSR accessions (Fig. 3b; Additional file 1: Tables S27-29). A comparison of average *z*-values for subgenomes A and C found no significant differences. The detected genomic regions under selection were annotated to determine genes located in those regions (Additional file 1: Tables S30-32). GO enrichment analysis for swede vs. non-swede accessions revealed significant overrepresentation of genes involved in cell division and expansion, environmental responses, and disease resistance (Additional file 1: Table S33). Analysis for East Asian OSR and all other OSR accessions pairwise comparison found significant enrichment for various genetic pathways including genes related to development, environment interaction, and abiotic stress responses (Additional file 1: Table S34). GO enrichment analysis for comparison of WOSR vs. SOSR accessions, detected enrichment for genes involved in reproductive growth and development, environmental interaction, and abiotic stress response in regions under selection (Additional file 1: Table S35). Since some inversions may be in linkage disequilibrium (LD) with insertions and deletions, or contain them, we also investigated the regions identified to be putatively under selection that overlapped with the candidate loci under selection identified for collective SVs. A total of 14, 9, and 7 candidate regions overlapped with those detected using collective SVs for the pairwise comparisons of swede vs. non-swede, East Asian OSR vs. all other OSR, and WOSR vs. SOSR accessions, respectively (Fig. 3b; Additional file 1: Tables S36-38). These findings suggest that the inversions have contributed considerably to intraspecific selective signatures resulting from intraspecific morphotype diversification and human selection in *B. napus*.

### Pangenome-wide gene PAV revealed association with morphological and intraspecific diversification

**Fig. 4:**
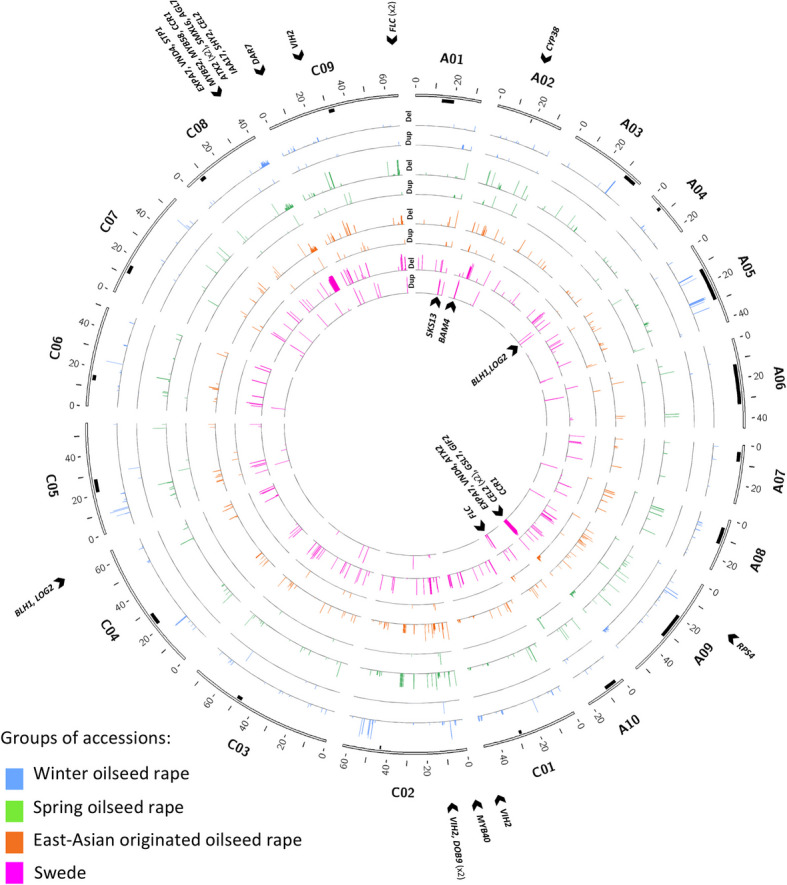
Overview of pangenome-wide gene presence-absence variations correlating with ecotype diversification in diverse accessions of *B. napus*. From outermost circles inwards, each pair of plots describes results for WOSR (blue), SOSR (green), East Asian OSR (orange), and swede/rutabaga (magenta) accessions. The distribution (*x*-axis) and percentage (*y*-axis, 0 to 100) of genes whose deletion or duplication correlated with each ecotype group are presented based on their presence in ≥ 70% of the target ecotype group and ≤ 30% of other ecotype groups. East Asian OSR accessions were not included as “other ecotypes,” as some accessions of this group clustered closely with WOSR and SOSR accessions in principal component analysis and phylogenetic analysis. The candidate genes that may correspond to ecotype diversification in deleted and duplicated regions are indicated outside and inside the circos plot, respectively. Plots were generated using Circos [105]

Genomic rearrangements, including large duplicated or deleted genomic segments, give rise to large-scale gene presence-absence variation. Analyzing the depth of reads aligned to the D10 reference genome for each accession revealed genomic segments of ≥ 10 kbp that were either duplicated or deleted. The chromosomes of subgenome C exhibited a higher number of large duplicated and deleted regions compared to those of subgenome A, with segmental deletions being more frequent than duplications. In total, 103,163 genes were affected by these large duplicated or deleted segments across the entire panel (Additional file 1: Table S39). From these, 829 genes were over-represented among either WOSR, SOSR, East Asian OSR, or swede accessions (≥ 70% in the target group and ≤ 30% in the other groups). Notably, in all swede accessions, large segmental deletions were observed on chromosomes C01, C02, C08, and C09, while segmental duplications were found on chromosome A09. The East Asian OSR and WOSR groups exhibited small deletions in all accessions on chromosomes C03 and C05, respectively (Fig. 4; Additional file 1: Table S40). Comparative analysis between the swede and non-swede groups highlighted genes located in duplicated and deleted regions, some showing more than one copy number change, that are potentially involved in cell wall development and expansion, lignin biosynthesis, and flowering. Specifically, large deleted segments in subgenome C, present in all swede accessions, contained one copy of *BnCEL2.C08* and *BnEXPA7.C08*, and two copies of *BnATX2.C08* and *BnFLC.C09*, which are orthologs of *Arabidopsis CELLULASE 2 (CEL2)*, *EXPANSIN A7 (EXPA7)*, *TRITHORAX 2 SET DOMAIN PROTEIN 30 (ATX2)*, and *FLOWERING LOCUS C (FLC)*, respectively. Additionally, large duplicated regions in most swede accessions carried two copies of *BnCEL2.A09* but just one copy each of *EXPA7.A09*, *BnATX2.A09*, and *BnFLC.A10*. These findings demonstrate that large chromosomal deletions and duplications are associated with the diversification of swede and non-swede groups, further supporting the role of large structural variants in the intraspecific diversification of *B. napus*.

### Pangenome-wide inversions are enriched for transposable elements

**Fig. 5:**
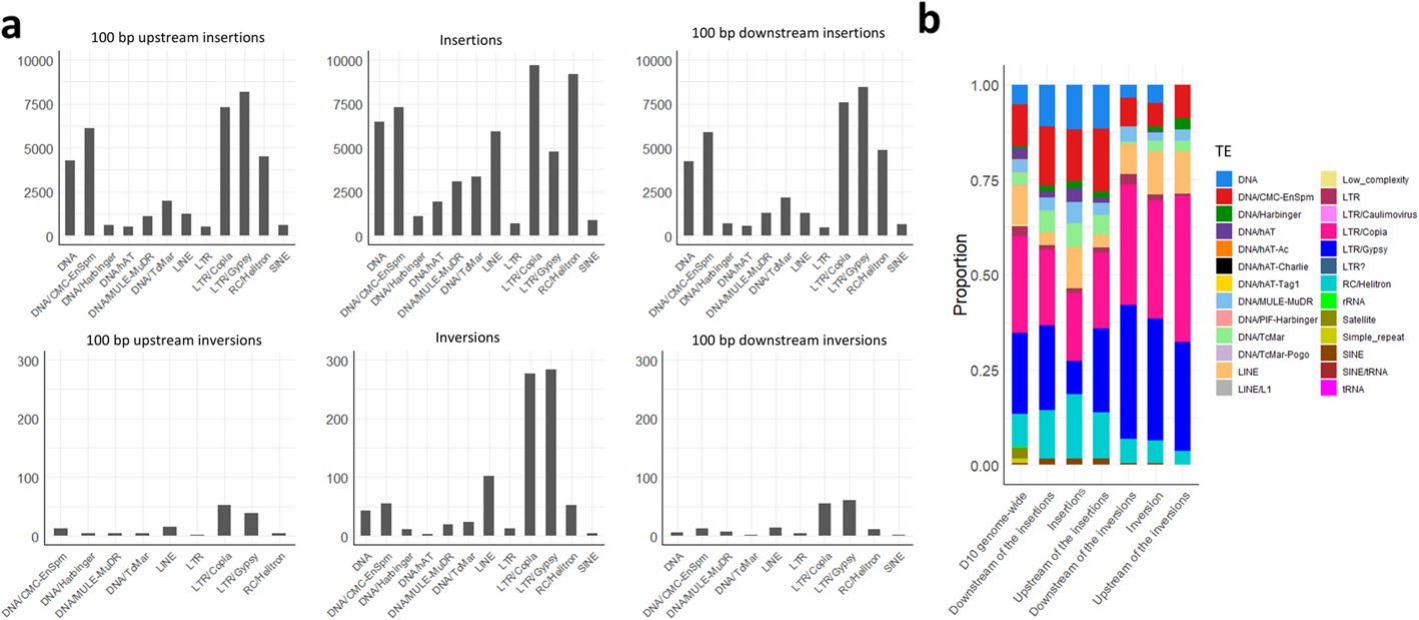
Overview of the distribution of transposable element (TE) families for insertion and inversion events in diverse accessions of *B. napus*. **a** Frequencies (*y*-axis) of different TE families (*x*-axis) in pangenome-wide insertion (top) and inversion (bottom) events. Bar plots show the frequencies of TEs observed within SVs, and in the 100-bp regions flanking SV breakpoints. **b** Proportion (*y*-axis) of different TE families in pangenome-wide insertion and inversion events in comparison to Darmore v10 genome [44] (*x*-axis). Each bar represents the relative abundance of various repetitive element categories, color-coded according to the legend on the right. These include DNA transposons (e.g., DNA/CMC-EnSpm, DNA/Harbinger, DNA/hAT, DNA/MULE-MuDR), retrotransposons (e.g., LINEs, SINEs), and LTR (Long Terminal Repeat) elements (e.g., LTR/Copia, LTR/Gypsy, LTR/Caulimovirus), as well as other elements including repetitive elements such as low-complexity regions, or unclassified DNA transposons

Out of 755,656 insertions, a total of 54,313 contained DNA sequences with partial or complete sequence identity (> 99%) with transposable elements (TEs). Sequence identity (> 99%) with TEs was found in regions upstream (− 100 bp) the breakpoints for 36,842 insertions, while 38,073 insertions had sequence identity with TEs downstream (+ 100 bp) of the breakpoints. For 1166 inversions, 890 contained sequences with partial or complete sequence identity (> 99%) with TEs. Total 136 inversions had sequence identity with TEs in regions upstream (− 100 bp) of the breakpoints, and 174 had sequence identity with TEs downstream (+ 100 bp) of the breakpoints. We identified 12 TE families. Insertions frequently contained LTR/Copia, RC/Helitron, CMC-EnSpm, and LINE elements, while their breakpoints had a higher presence of LTR/Gypsy, LTR/Copia, and CMC-EnSpm elements. Sequences within inversions and their breakpoints exhibited a higher presence of LTR/Gypsy and LTR/Copia elements (Fig. 5).

### Abundant repetitive sequences at breakpoints of collective SVs and inversions

To investigate the mechanisms involved in the formation of insertions, deletions, and inversions, genomic regions flanking 100 bp upstream and downstream of putative breakpoints were searched for sequence motifs, as well as direct or inverted repeats. We separately analyzed insertion and deletion events from the collective SVs. Deletions and insertions were divided into different size groups ranging from 30 bp to 30 kbp to assess whether different sequence motifs were associated with different size groups. This analysis revealed the presence of direct repeats (including microsatellites), and inverted repeats at the breakpoints of both ends for all investigated categories, suggesting a non-random distribution. The results showed significant enrichment of poly-A or poly-T stretches and GC-rich palindromic sequences. Poly-A or poly-T sequences were abundant across events in all categories (Additional file 2: Fig. S2). For inversion events, different patterns of GC-rich palindromic sequences were found at the breakpoints (Additional file 2: Fig. S3).

### Saturation analysis indicates near-complete pangenomic coverage

The Saturation curve analysis was performed using genes overlapping pangenome-wide SNPs. The gene accumulation curve showed a gradual increase in the number of unique genes with each additional accession, with the rate of new gene discovery declining as more genomes were added. This trend indicates a near-saturation of the *B. napus* pangenome with the current selection of accessions. The curve began to plateau after approximately 50 accessions, suggesting that most gene content diversity is captured within the current set of 94 accessions (Additional file 2: Fig. S4).

### Gene flow analysis captures historical gene flows within *B. napus*

**Fig. 6:**
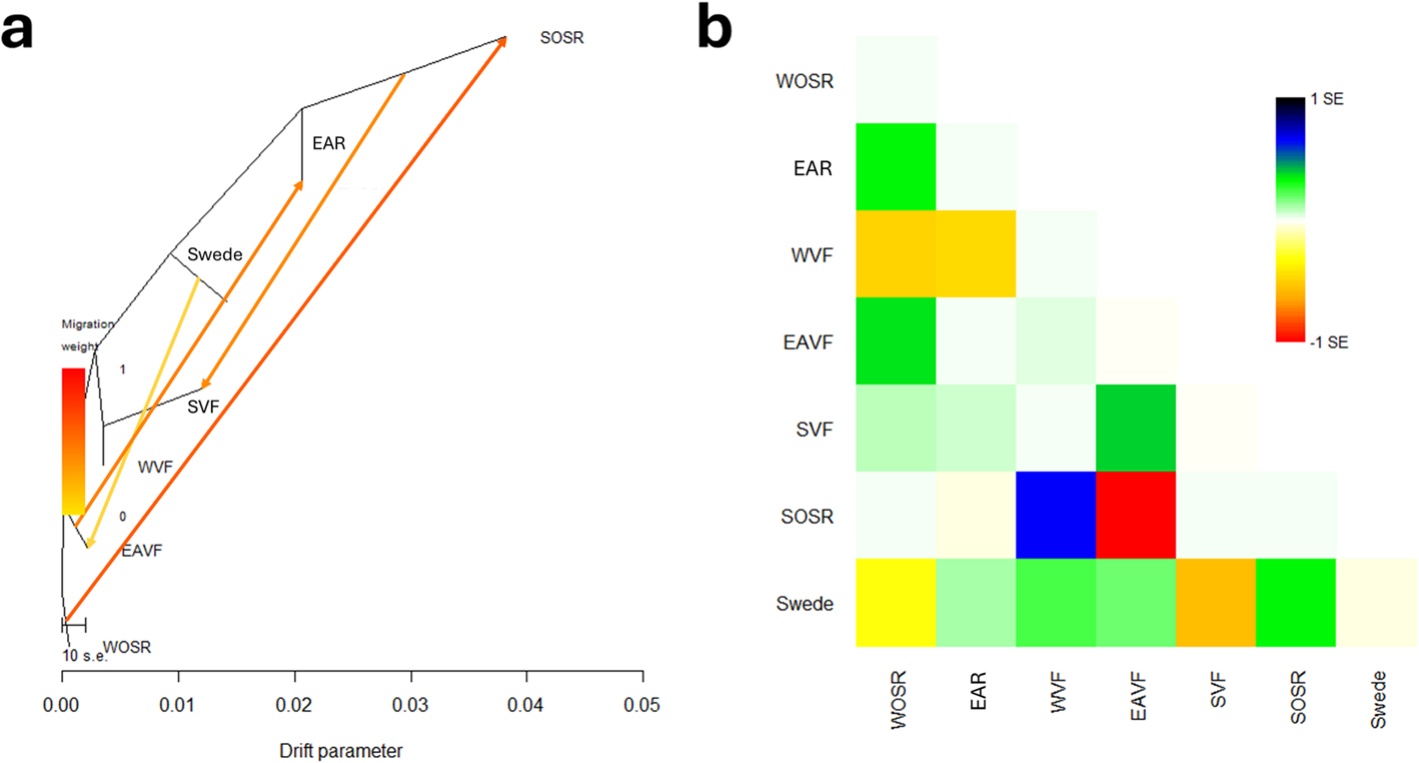
Patterns of gene flow among seven *B. napus* groups. **a** Maximum-likelihood population tree inferred using TreeMix with four migration edges and SV block size of 1000. WOSR (winter oilseed rape) was specified as the root. Migration edges are shown as arrows, with color indicating migration weight (yellow to red scale, with red indicating higher migration weight). The *x*-axis reflects the drift parameter, which is proportional to genetic drift experienced by each population. **b** Heatmap of residuals from the TreeMix model with four migration edges. Each cell represents the scaled residual covariance between pairs of groups, with warmer colors (toward red) indicating pairs with more covariance than expected and potentially missed gene flow, and cooler colors (toward blue) indicating pairs with less covariance than expected and possibly overestimated relatedness. Residuals are normalized by the standard error (SE), with the color scale indicating ± 1 SE. Abbreviations of the groups are as follows: WOSR, Winter oilseed rape; WVF, Winter vegetable/fodder rape; SOSR, Spring oilseed rape; SVF, Spring vegetable/fodder rape; EAR, East Asian oilseed rape; EAVF, East Asian vegetable/fodder rape; Swede

To elucidate patterns of gene flow among seven *B. napus* groups, we performed gene flow analysis using collective SVs and modeled four migration events using TreeMix v1.13 with a block size of 1000 SVs. The residual plot showed that majority of covariance among groups were explained, indicating that the model with four migration edges captures relationships among the majority of each two groups. Model selection using the OptM package confirmed four migration edges as the optimal number. The resulting maximum likelihood tree revealed gene flow directions including two strong gene flow signals from WOSR to SOSR, and from SOSR to spring vegetable/fodder type. Notably, two additional gene flow events were detected from Swede to East Asian vegetable/fodder type, and from East Asian vegetable/fodder to East Asian OSR (Fig. 6).

## Discussion

In this study, we surveyed the SV landscape of 94 ecogeographically diverse *Brassica napus* accessions using long-read sequencing data and reference-guided genome assemblies. A *B. napus* pangenome SV Atlas was constructed describing distribution and frequencies for collective SVs, inversions, and gene PAV, capturing species-wide insertions, deletions, inversions, and gene presence-absence variations across the divergent *B. napus* morphotypes. We found that the collective SVs and inversions affect up to 15.14 and 11.47% of the genome, respectively. The pangenome-wide gene PAV indicated that 38.88% of *B. napus* genes were affected by large segmental chromosomal deletions in at least two accessions, and 78.97% by large segmental duplications. Using this diverse species-wide panel of accessions, our findings demonstrated that a broad spectrum of structural variant types is linked to intraspecific diversification in *B. napus*, emphasizing their collective significance in evolution and breeding of this important, recent allopolyploid crop species. Candidate loci under selection based on the distribution and frequency of collective SVs and inversions, as well as gene PAV linked to intraspecific diversification, were predominantly located outside centromeric regions and in chromosome arms, which is consistent with the expected locations of chromosomal crossover [47] and the predominance of coding genes in *B. napus* [4]. Regions under selective pressure for intraspecific morphotype diversification contained putative genes associated with various adaptation mechanisms, such as flowering time, cell wall expansion, organ development, environmental response, abiotic stress response, and disease resistance, as previously described in studies exploring SNPs, InDels, CNVs, and homoeologous chromosomal exchanges across different *B. napus* ecotypes [4, 15, 20, 28, 30, 31].

The accuracy of SV identification is highly dependent on the quality and completeness of the reference genome assembly used. In this study, we used the gold-standard Darmor-bzh v10 reference genome [44] for SV detection. While pangenome references provide a more comprehensive representation of species-wide diversity, at the time of conducting this research, the computational tools and SV-calling algorithms tailored for pangenome-based analysis were still in early stages of development, particularly for complex polyploid genomes such as *B. napus*. Furthermore, using a reference pangenome can complicate the detection of accession-specific insertions, as sequences may not be flagged as novel as they are already in the reference pangenome. In contrast, a single high-quality reference genome offers a consistent baseline for detecting various SV types with high resolution and confidence and enables the use of robust and well-validated SV detection tools. Although some SVs detected in our study overlap with those reported in previous pangenome-based analyses such as Song et al. [40], a detailed comparative analysis of SVs would require harmonizing reference structures, coordinate systems, and variant definitions, which remains a valuable area for future research.

Principal component and phylogenetic analyses based on collective SVs and inversions distinguished the major ecotypic groups including WOSR, SOSR, East Asian OSR, and swede/rutabaga, indicating that the selected panel of 94 accessions captures the species-wide known population structure of *B. napus*. We observed that a number of accessions did not align with morphotype classifications. This likely reflects the complex breeding history of *B. napus*, including historical outcrossing, selection, and possible undocumented introgressions. While de novo structural variants could contribute to this pattern, our current data do not support such a mechanism. Therefore, the observed inconsistencies highlight the limitations of phenotype-based classifications in capturing the underlying genomic diversity, as these classifications are based largely on agronomic traits and environmental adaptation rather than genomic relationships. Additionally, the saturation curve analysis using pangenome-wide SNP data, revealed a curve that began to plateau after approximately 50 accessions, which supports the sufficiency of our set of accessions to capture the pangenomic diversity of *B. napus*.

Phylogenetic analyses based on collective SVs and inversions showed a closer clustering of swede and East Asian OSR accessions to *B. rapa.* During the adaptation of rapeseed introduced from Europe to East Asia, repeated introgressions from Asian *B. rapa* into Asian *B. napus* cultivars have been documented [18, 48]. Furthermore, evidence of interploidy gene flow from European turnip into swede has been reported [20]. It is accepted that *B. rapa* is the maternal progenitor of rapeseed [15]. Although the initial ancestral parents are unknown, this allopolyploid species is extremely recent compared to the 4.6 million years since *B. rapa* and *B. oleracea* diverged [49]. Nevertheless, introgressions from diploid progenitors and swede [22] during the adaptation history of OSR, along with the fact that no wild *B. napus* exists [15], makes it complicated to trace its order of divergence and ancestral lineage [21].

Our study employed insertions, deletions, inversions, and gene presence-absence variations for a species-wide investigation of subgenome asymmetry, as well as an analysis of candidate loci under selection in *B. napus*. Previous studies utilized other types of genetic variations, such as SNPs [4, 15, 20, 27, 28], SSRs [29], CNVs [30], and homoeologous chromosomal exchanges [31]. Based on structural variations, we demonstrated an unbalanced distribution of SVs, and an asymmetrical selection pattern between *B. napus* subgenomes. We observed a higher number of insertions and deletions in subgenome A compared to subgenome C across the panel. Furthermore, there were more genes with SVs in coding sequences (CDS) and untranslated regions (UTRs) associated with R genes in subgenome A compared to C, while flowering-time-related genes were more prevalent in subgenome C than in subgenome A. The pangenome-wide distribution and frequency of collective SVs revealed stronger putative selection signals in subgenome C compared to subgenome A when comparing swede vs. non-swede, and WOSR vs. SOSR, with the greatest difference observed for swede vs. non-swede. The observed asymmetry between the *B. napus* subgenomes is a complex phenomenon attributed to inherent characteristics of the progenitor species prior to hybridization, along with the influence of breeding procedures that utilized progenitors or closely related crops as donors. A previous study using a diversity panel representing variability in Chinese semi-winter rapeseed reported a higher rate of recombination and genetic diversity in subgenome A [50], likely reflecting past crossings between *B. rapa* and *B. napus* and the better viability of the resulting hybrid seeds compared to attempts to cross *B. oleracea* and *B. napus* [51, 52]. Another study, which focused mainly on accessions from Asia, investigated divergence in gene function and differential selection pressures between the two subgenomes post-polyploidization, reporting stronger selective sweep signatures in subgenome C for (East Asian) semi-winter vs. winter, and spring vs. winter rapeseed. This study highlighted that during the breeding process from original *B. napus* to landraces, asymmetrical subgenomic selection of genes led to ecotype change. This included improvements in *B. napus* with respect to adaptation to environmental variation and oil accumulation through A subgenome-specific selection on defense-response genes and triglyceride biosynthetic genes, respectively [15]. Similarly, another study focusing on mostly Asian accessions reported more genes associated with plant defense and oil accumulation in subgenome A, whereas developmental-related genes, including flowering-time-related genes, were more prevalent in subgenome C compared to A [53]. Gene expression studies have also noted biases depending on the history of the plant material or tissue used. Some reported an expression bias toward subgenome A [54], while others observed a bias toward subgenome C [55]. Although the regions identified in this study suggest potential targets of selection, including those overlapping genes with putative roles in the diversification of the compared ecotype groups, they should be interpreted as putative candidates rather than confirmed selective sweeps. The inclusion of additional complementary statistical approaches such as analyses of nucleotide diversity ratios and haplotype-based methods, would enhance the robustness of these findings which could be further investigated in future studies to confirm these signals of selection as selective sweeps. Furthermore, an *F*_ST_ outlier often reflects extreme reductions in polymorphism at a specific locus. These reductions can result from selective sweeps acting directly on adaptive traits, or they may arise indirectly due to linked selection, such as background selection against deleterious mutations [56]. When interpreting *F*_ST_ outliers in pericentromeric regions which are known to have low recombination rate [47], the possibility that elevated *F*_ST_ values may reflect increased linkage disequilibrium rather than true signals of selection, should also be considered.

When comparing swede vs. non-swede, East Asian OSR vs. all other OSR, and WOSR vs. SOSR accessions, our findings revealed that SVs impacted genes with putative roles in morphotype development, disease resistance, responses to environmental, and abiotic stress. These included genes with SV-carrying variants coinciding with the identified candidate loci under selection or genes affected by large segmental presence-absence variations. For swede, the identified genes were putatively involved in storage organ formation, cell division and expansion, meiosis, and environmental responses and disease resistance, highlighting morphotypic differences between swedes and non-swedes. For East Asian OSR vs. all other OSR, regions under selection contained genes linked to environmental response, disease resistance, and abiotic stress response, aligning with historical records suggesting that rapeseed underwent breeding after its introduction from Europe to East Asia to adapt to new environments [15]. Similarly, in the WOSR vs. SOSR comparison, genes putatively involved in environment interaction, stress response, disease resistance, and flowering time were identified. The role of SVs in differentiating WOSR and SOSR, particularly for flowering time genes such as *BnFLC.A03* and *BnFLC.A10*, has been well-documented [40, 57]. Intragenic SVs can affect gene variants differently depending on their location within coding and untranslated regions, as well as their size and number. However, the overlap of SV-carrying gene variants with the identified candidate loci under selection can imply their importance for further studies. Large chromosomal segment duplications and deletions also distinguished swede from OSR, particularly in subgenome C. For instance, gene presence-absence variation analysis found genes that are reported to have higher expression in swede compared to rapeseed during swollen hypocotyl formation [31], such as homoeologous gene pair *BnBLH1.A04 *and* BnBLH1.C04* which were located in a duplicated and deleted segment respectively*.* Results also revealed two copies of *BnFLC.C09* and *BnATX2.C08*, both putative flowering time inhibitor genes, and one copy of *BnCEL2.C08*, a putative cell wall development and expansion gene, in a deleted segment. Meanwhile, one copy each of *BnFLC.A10* and *BnATX2.A09*, and two copies of *BnCEL2.A09* were found within duplicated segments, offering a more detailed perspective compared to previous studies focused solely on flowering time genes [30].

Gene flow analysis revealed a migration event from WOSR to SOSR which aligned with the reports that spring oilseed rape diverged from winter oilseed rape through breeding and adaptation processes [15]. Additionally, the analysis identified gene flow from Swede to East Asian vegetable/fodder type, as well as from East Asian vegetable/fodder type to East Asian OSR. This is in line with what is documented about the gene flow between European gene pools and East Asian *B. napus*, including semi-winter accessions, and is further supported by studies reporting repeated introgressions from Asian *B. rapa* into Asian *B. napus* cultivars [18, 48]. Our phylogenetic analysis also demonstrated a closer clustering of Swede and East Asian OSR accessions with *B. rapa*, suggesting a close genetic background.

To ensure high-confidence SV detection, we implemented a stringent multi-step filtering pipeline based on a previously validated long-read SV detection framework by Chawla et al. [46]. These steps were designed to mitigate the error rate associated with long-read sequencing platforms such as Oxford Nanopore [58] and reduce false positive SV detection, to retain the high-confidence SV calls. Although this provides a robust dataset, additional validation of selected SVs through experimental approaches or alternative data remains to be pursued in future studies. We focused on homozygous SV loci and excluded heterozygous SVs to ensure confident variant detection. The accessions used were inbred lines, as previously described by Bus et al. [29], and were further subjected to four generations of selfing to increase homozygosity. Given the polyploid nature of *Brassica napus*, this strategy helps minimize false positives caused by misaligned homoeologous regions and also reduces potential bias from any residual heterozygosity. Although heterozygous SVs may include functionally important variations, we prioritized confident SV identification to avoid overinterpreting potentially artifactual calls. Future studies may incorporate phased SV analysis or haplotype-aware genotyping to more accurately capture heterozygous SVs.

Structural variant (SV) calling methods based on read alignment [59] allow for the filtering of SVs by read depth, which enhances the accuracy of SV detection. However, despite advancements in long-read sequencing technology which have increased read length, this approach becomes less efficient for detecting large SVs that exceed the read length. To address this limitation, we used a reference-guided genome assembly approach to detect inversions larger than 1 Mb. Nonetheless, this approach is still limited in detecting more complex SVs, such as translocations or very large inversions, as the alignment procedure assumes a linear and collinear relationship with the reference genome.

We identified inversion hotspots within or near centromeric regions throughout the *B. napus* panel, consistent with reports that inversions in the Arabideae tribe are often enriched in these regions [60]. Such inversions can contribute to the suppression of meiotic crossovers, preserving the structural integrity of the centromeres and maintaining their proper function during cell division [60, 61], which reduces the influence of selection in these regions [62].

Analysis of transposable element (TE) content in inversions showed an enrichment to 76.33% compared to the average TE content of 54% observed in the Darmor v10 genome [44]. This suggests a potential role for TEs including TE-mediated genomic rearrangements in the origin of many inversions [63]. Long terminal repeat (LTR) Copia and Gypsy elements were the most abundant TE families in inversion sequences and inversion breakpoints. Similar findings have been reported in a pangenome inversion index constructed for rice [64]. Differences associated with various TE families has been reported in the sequence of insertions located in similar position in different genotypes, indicating connection with diversification of *B. napus* [40]. Therefore, we considered both position and similarity of sequences, when merging SVs from accessions throughout the panel. We investigated abundance of TE content in insertions which showed 7.2% of insertions had high sequence similarity to various TE families. On the other hand, our analysis of SV breakpoint flanking sequences revealed enrichment for direct and inverted repeats, poly-A/T tracts, and GC-rich palindromic motifs, which suggests that a significant proportion of insertions arise from non-TE mechanisms. Specifically, observed patterns are consistent with insertional events driven by double-strand break repair pathways, such as non-homologous end joining and microhomology-mediated end joining, which can incorporate filler DNA fragments at repair sites [65, 66]. Other mechanisms reported as the origin of insertions include tandem and segmental duplications formed via non-allelic homologous recombination [67], as well as homoeologous recombination between the A and C subgenomes of *B. napus*, which contribute to non-reciprocal segmental insertions [5, 68]. It is also possible that some insertions derive from highly diverged or degenerate TEs that were not detected by current annotation tools.

We investigated inversions and their connection to intraspecific diversification and identified large inversions that are prominent in swedes and SOSR. Our findings showed that the strength and frequency of putative selection signals based on inversions increased, on average, outside centromeric regions and toward paracentric regions in all pairwise comparisons. This aligns with the reported role of inversions in species divergence and adaptation, by potentially preserving beneficial gene combinations while reducing the probability of crossover within the inverted region. Large inversions and large PAV are known to influence occurrence of recombination [69]. Large segmental chromosomal PAV and inversions distinguished swedes from non-swede accessions. For example, segmental deletions and duplications larger than 10 kbp in chromosomes A09, C01, C02, C08, and C09 differentiated swedes from all OSR ecotypes. Also, a deletion was found in all accessions of East Asian OSR in chromosome C03 and another deletion in all WOSR accessions in chromosome C05. These duplications or deletions may have arisen from unbalanced gametes formed due to meiotic recombination in paracentric regions [70], leading to non-reciprocal chromosomal exchanges. Individual swede accessions were reported earlier to carry a higher level of homoeologous chromosomal exchanges compared to OSR, particularly between choromosomes C01-A01, C08-A09, and C09-A10, and mostly in the direction of A subgenome segments replacing those of C [31]. This could be detected as deleted segments in subgenome C chromosomes such as C01 and C08 [4]. By demonstrating that these and other large SVs are specific to particular crop morphotypes or ecotypes, we show that such variants were likely a key factor in the formation of novel phenological and physiological variation that was selected by humans during evolution of *B. napus* crops.

## Conclusions

In conclusion, our study provides a comprehensive survey of the pangenome-wide structural variations in the important crop *B. napus* and demonstrates that a broad spectrum of SVs is associated with intraspecific morphotype and ecotype diversification. The study provides valuable insights into SVs associated with selection signals throughout the evolution and breeding of *B. napus*, as well as the underlying genes and genetic elements in affected chromosome regions. This comprehensive analysis of the SV landscape offers a valuable resource for further improving *B. napus* as a crop, for example by implementing SVs in breeding. Additionally, larger SVs, such as inversions and segmental deletions and duplications, can shed light on how these variants have influenced patterns of genomic recombination within each ecotype or subspecies, and help overcome chromosome regions which are recalcitrant for recombination.

## Methods

### Plant material

Aiming for species-wide detection of structural variations in *B. napus*, we selected 94 genetically and ecogeographically diverse accessions broadly representing the genetic diversity present in the ERANET-ASSYST diversity set, a species-wide panel of over 500 inbred lines previously described by Bus et al. [29]. The selected accessions originated primarily from Europe and East Asia, the main centers of origin for modern *B. napus* crop types, along with key oilseed and swede cultivars bred in North America and Australasia. Prior to sequencing, all accessions were maintained through controlled self-pollination for at least four generations in Plant Breeding Department at Justus Liebig University Giessen, Germany. Ecotype classification followed Bus et al. and included WOSR (22 accessions), SOSR (23 accessions), East Asian OSR (22 accessions), and swede/rutabaga (17 accessions), along with Asian *B. napus* vegetable forms and fodder rape accessions [29]. Detailed information about morphotypes, ecotypes, and geographic origins for each accession is presented in Additional file 1: Table S1. Long-read sequencing data for two accessions of *B. rapa* (*B. rapa* ssp. *rapa* and *B. rapa* ssp. *broccolieto*) [71], and two accessions of *B. oleracea* (Genebank/cultivar IDs HRIGRU007343 and HRIGRU007338) [35], as well as genome assemblies of *B. rapa* Z1 (yellow sarson) and *B. oleracea* HDEM (broccoli) [72], were downloaded from public resources to represent the diploid progenitors.

### High molecular weight DNA isolation and long-read sequencing

Three plants from each accession were grown in a greenhouse to generate tissue, one plant intended for sampling and two as back up. Fully expanded young leaves were collected, immediately deep-frozen in liquid nitrogen, and stored at − 80 °C for DNA extraction. High molecular weight (HMW) genomic DNA was extracted from 2.5 g of the two youngest leaves from a single plant of each accession using the NucleoBond HMW DNA extraction kit (Macherey–Nagel, Germany). The kit protocols were followed according to the manufacturer’s instructions. DNA quality was assessed via 0.5% agarose gel electrophoresis (80 V, 0.5 × TBE, 60 min), a UV/Vis spectrophotometer (NanoDrop Technologies, USA), and a Qubit fluorometer using the Qubit dsDNA BR assay kit (Life Technologies, USA). Long-read DNA sequencing using Oxford Nanopore Technology was conducted by the DFG NGS Competence Center Tübingen (NCCT, Tübingen, Germany). HMW DNA was further qualified using the FP-1002 Genomic DNA 165-kbp kit for FEMTO Pulse systems (Agilent Technologies, USA). Library preparation was performed using the native ligation sequencing kit (SQK-LSK109; Oxford Nanopore Technologies), followed by sequencing using Oxford Nanopore Technology PromethION R9.4.1 flow cells. DNA fragments smaller than 25 kbp were progressively depleted before library preparation using the Short Read Eliminator kit (Circulomics, USA) to enhance read length and sequencing output. Base calling was performed using Guppy Basecaller v.5.1.12 and 6.1.5 (Oxford Nanopore Technologies) with the dna_r9.4.1_450bps_hac_prom model.

### Constructing reference-guided genome assemblies

De novo contig assembly was performed using the filtered long reads as input with Flye v2.9-b1768 [73]. The de novo assembled contigs were scaffolded based on the Darmor-bzh v10 (D10) reference genome [44] using Ragtag v2.1.0 [74]. N50, assembly size, and BUSCO scores were measured, and assembly statistics were calculated with Assembly-stats v1.0.1 [75]. The completeness of the assemblies was evaluated using BUSCO v5.7.1 [76] with the embryophyte_odb10 lineage dataset.

### Aligning long reads to reference genome and determining pangenome-wide distribution and frequency of collective SVs

To remove any artificial bias from the raw long reads, they were filtered using NanoFilt v2.8.0 [77] with the following parameters: minimum read length of 5000 bp, minimum quality score of 10, and removal of potential adapter or error-prone regions by trimming 60 bases from both the 5′ and 3′ ends (parameters: -l 5000 -q 10 --headcrop 60 --tailcrop 60). The quality of the filtered data was assessed using NanoPlot v1.40.0 [77], and genome-wide coverage was estimated per sample. The trimmed raw reads were aligned to the D10 reference genome [44] using Minimap2 v2.24-r1122 [78] with ONT-specific presets (parameters: -x map-ont --MD -a). Only high-quality alignments were retained using the view function from SAMtools v1.10 [79] (parameters: -bS -F 4 -h -q 50), which excluded unmapped reads and those with mapping quality below 50.

Structural variant (SV) calling was based on the methodology previously validated by Chawla et al. and Yildiz et al. [46, 80], with addition of modifications that included an elaborate pipeline comprising multiple SV callers and rigorous filtering steps. Filtered aligned reads were used as input for SV calling. SVs were called independently using Sniffles v2.0.6 [81] (parameters: --minsupport $SV_minsupport --minsvlen 30) and CuteSV v1.0.13 [82] (parameters: -s $SV_minsupport --genotype -l 30 --max_cluster_bias_INS 100 --diff_ratio_merging_INS 0.3 --max_cluster_bias_DEL 100 --diff_ratio_merging_DEL 0.3 --retain_work_dir --report_readid). The parameter "$SV_minsupport" was defined as 40% of the sequencing data coverage per sample. This allowed to ensure minimum read support proportional to data coverage. To ensure SVs reflected true variation and not misalignment in duplicated homoeologous regions, only homozygous SV loci (allele frequency ≥ 0.9) with precise breakpoints were retained for each SV caller. We recognize the limitations of this choice regarding filtering out possible functionally important heterozygous SVs; however, focusing on homozygous SVs minimizes noise from polyploid genome structure, where homeologous exchanges and alignment ambiguity can inflate heterozygous SV calls. While this may exclude biologically relevant heterozygous SVs (e.g., masked deleterious alleles), this trade-off was necessary to ensure high-confidence variant discovery in a structurally complex genome.

To reduce false positives, only the filtered SVs detected by both callers were retained by merging outputs using Survivor v1.0.7 [83] (parameters: 1000 2 1 1 −1 −1), which retained homozygous SV loci with precise breakpoints detected by both callers. These filtered SVs were then used for a re-genotyping (force-calling) step using Sniffles, followed by additional filtering of the re-genotyped SVs (parameters: PASS, QUAL ≥ 50, GQ ≥ 50, DV ≥ 12).

Due to the complexity of the *B. napus* genome, particularly the presence of homoeologous genomic regions and potential exchanges between subgenomes A and C, several scenarios were considered during the filtering of the SV call sets, including reciprocal and non-reciprocal chromosomal homoeologous exchanges. Notably, non-reciprocal homoeologous exchanges, where two copies of a chromosomal segment from one subgenome exist while the corresponding segment in the other subgenome is deleted, can be detected as duplicated and deleted segments by analyzing the coverage of long reads aligned to the reference genome. A read coverage calculation pipeline detailed by Stein et al. [68] used the aligned bam files to estimate the read coverage, and the areas with lower read coverage were considered as deleted chromosomal segments. To filter-out the SVs that overlapped these segments (≥ 10 kbp), the following procedure was applied using this pipeline with modification: Aligned reads were used to calculate coverage across chromosomes using the bamtobed and genomecov functions from BEDTools v2.30.0 [84], which served as input for the modified deletion-duplication pipeline [68]. Outlier regions with coverage above 150 were discarded, and segments ≥ 10 kbp with coverage deviating by at least one standard deviation below the mean were considered as deleted segments. The resulting BED file was used as input for the intersect function from BEDTools to remove SVs from the call set that overlapped with large deleted segments.

Finally, SVs from all accessions were merged using Jasmine v1.1.5 [85] (parameters: max_dist = 1000 min_support = 1 min_seq_id = 0.9 --nonlinear_dist --output_genotypes --normalize_type). This software allows to account for sequence identity to differentiate SVs based on both position and sequence similarity. For the merging process, variants within 1000 bp of each other were considered the same, provided they shared at least 90% sequence identity, and single-sample variants were also retained. Summary of workflow steps, tools, and parameters is presented in Table 1. For validation, SVs were further visualized with the Integrative Genomics Viewer (IGV) tool [86] and compared to previously published SVs. Long-read sequencing data for *B. rapa* and *B. oleracea* were aligned to their respective D10 subgenomes, A and C.

**Table 1:** Summary of workflow steps, tools, and parameters, for SV detection including collective SVs and inversions

Pipeline	Step	Tool/Software	Parameters
Filtering raw reads	Filtering raw reads	NanoFilt v2.8.0	-l 5000 -q 10 --headcrop 60 --tailcrop 60
	Quality check of filtered reads	NanoPlot v1.40.0	Default
Detecting collective SVs	Alignment of trimmed reads to reference genome	Minimap2 v2.24-r1122	-x map-ont --MD -a
	Filtering alignments	Samtools v1.10	view function, -bS -F 4 -h -q 50
	SV calling	Sniffles v2.0.6	--minsupport $SV_minsupport --minsvlen 30
	SV calling	CuteSV v1.0.13	-s $SV_minsupport --genotype -l 30 --max_cluster_bias_INS 100 --diff_ratio_merging_INS 0.3 --max_cluster_bias_DEL 100 --diff_ratio_merging_DEL 0.3 --retain_work_dir –report_readid
	Filtering called SVs	custom script	Allele frequency ≥ 0.9, precise breakpoint
	Retaining SVs called by both Sniffles and CuteSV	SURVIVOR v1.0.7	1000 2 1 1 −1−1
	SV re-genotyping	Sniffles v2.0.6	Default
	Filtering re-genotyped SVs	custom script	PASS, QUAL ≥ 50, GQ ≥ 50, DV ≥ 12
	Removing SVs that overlapped with large deleted segments (step1: calculate coverage across chromosome using aligned reads, detect large deleted segments (≥ 10 kbp))	Modified pipeline based on Stein et al	
	Removing SVs that overlapped with large deleted segments (step2: remove SVs that overlapped with deleted segments)	BEDTools v2.30.0	intersect function, removing SVs overlapping deleted segments (≥ 10 kbp)
	Final SV merging	Jasmine v1.1.5	max_dist = 1000 min_support = 1 min_seq_id = 0.9 --nonlinear_dist --output_genotypes --normalize_type
Detecting inversions	Genome Assembly Alignment	Minimap2 v2.24-r1122	-ax asm5 --eqx --cs -c --MD
	Inversion detection	SyRI v1.6.3	-F S --nosnp --tdgaplen 500,000
	Inversion Merging	Jasmine v1.1.5	max_dist = 1000 min_support = 1 min_seq_id = 0.9 --nonlinear_dist --output_genotypes --normalize_type
	Filtering inversions	Filtering (custom script/workflow)	PASS status, < INV > tags

### Annotation of putative functional SVs

To annotate putative functional SVs, the intersect function from BEDTools v2.30.0 [84] was used to identify overlaps between SV events and gene models in the GFF file of the D10 reference genome. Araport11 *A. thaliana* representative gene model complementary DNA (cDNA) sequences [87] from TAIR were downloaded to create a local database using BLASTn [88]. Functional annotation of genes was carried out by aligning the cDNA sequences of the D10 reference genome against the local database (parameters: -evalue 1e-4 -perc_identity 90 -ungapped -max_target_seqs 1 -max_hsps 1). A list of *Arabidopsis* flowering time regulators from the Flowering Interactive Database (http://www.phytosystems.ulg.ac.be/florid) [89] and a list of putative R genes for *B. napus* published by Dolatabadian et al. [90] were used to facilitate gene function annotation.

### Detecting inversions using reference-guided genome assemblies and determining pangenome-wide distribution and frequency

Inversions larger than 30 bp were detected using the 94 reference-guided genome assemblies following the approach detailed by Zhou et al. [64], with modifications, including the use of Minimap2 as the aligner [91]. In summary, each genome assembly was aligned to the D10 reference genome using Minimap2 v2.24-r1122 (parameters: -ax asm5 --eqx --cs -c --MD). Regions of synteny and inversions were identified using SyRI v1.6.3 [59] (parameters: -F S --nosnp --tdgaplen 500,000), and inversions were merged using Jasmine v1.1.5 [85], with filtering applied to the aligned sequences for a minimum identity of 90% (parameters: max_dist = 1000 min_support = 1 min_seq_id = 0.9 --nonlinear_dist --output_genotypes --normalize_type). SV events marked as < INV > and with a "PASS" status were retained. Summary of workflow steps, tools, and parameters is presented in Table 1. Inversions correlating with ecotype diversification for WOSR, SOSR, East Asian OSR, and swede/rutabaga accessions were separated into subsets based on the following criteria: ecotype of interest ≥ 70%, other ecotypes ≤ 30%, as over-represented inversions. The under-represented inversions were determined by ecotype of interest ≤ 30%, other ecotypes ≥ 70%. East Asian OSR accessions were not included as “other ecotypes” because some accessions in this group clustered closely with WOSR and SOSR accessions in principal component and phylogenetic analyses. The filtering was performed in R [92]; therefore, limitations in floating-point precision might occur. The assemblies of *B. rapa* and *B. oleracea* was aligned to their respective D10 subgenomes, A and C. To assess the statistical significance of over-represented and under-represented inversion events across ecotypes, Fisher’s exact test was applied to each inversion–ecotype combination. For each test, a 2 × 2 contingency table was constructed to compare the number of accessions with and without the inversion in the ecotype of interest versus all other ecotypes. From this, we derived *p*-values and odds ratios to quantify both significance and direction of association. The *p*-values were adjusted after applying the Benjamini–Hochberg correction. An odds ratio > 1 indicated significant enrichment of the inversion in the ecotype, while an odds ratio < 1 indicated depletion. The associations with adjusted *p*-value < 0.05 were considered statistically significant.

### Pangenome-wide SNP detection

Filtered aligned reads were used as input for SNP calling. SNPs were called using Clair3 v0.1-r12 [93] (parameters: --platform = ont --model_pathr941_prom_hac_g360 + g422 --include_all_ctgs --call_snp_only --haploid_sensitive). The detected SNPs were filtered using view function from BCFtools v1.17 [79] (parameters: PASS, QUAL ≥ 20, DP ≥ 14, AF ≥ 0.90). The BED file presenting the large deleted segments ≥ 10 kbp was used to remove SNP from the call set that overlapped with large deleted segments using the same pipeline described for filtering collective SVs. Then, SNPs were merged using merge function from BCFtools.

### Chromosomal distribution of pangenome SV hotspots

For an overview of distribution and localization of SV hotspots, we performed a 200-kbp fixed-window analysis across all chromosomes using the coverage function of BEDTools. The top 30% of windows with the highest frequency of SV start coordinates were defined as hotspots, separately for each subgenome. To compare subgenomes A and C, the number of events per each 200 kbp were standardized by percentage for whole genome and compared using one-tail Student’s test (*α* = 0.01).

### Phylogenetic analysis and principal component analysis

For phylogenetic analysis, SVs were encoded as binary presence/absence data for each accession, with values indicating whether a given SV was detected relative to the D10 reference genome. Then, the merged SV events for all accessions were used to construct maximum likelihood (ML) trees using IQ-TREE v2.2.0.3 [94], employing models specifically designed for binary data. Model selection was performed automatically based on the Bayesian Information Criterion (BIC), ensuring the best-fitting model was chosen for each dataset. For the collective SVs dataset, the BIC model GTR2 + FO + G4 was selected, and for inversions the model was GTR2 + FO + ASC + G4 (https://iqtree.github.io/doc/Substitution-Models). To further improve model robustness and biological relevance, the collective SVs dataset was filtered to include only intergenic SVs using the intersect function in BEDTools v2.30.0 [84], minimizing the impact of human selection on coding regions. Additionally, 25% of each chromosomal end was excluded to avoid SVs in subtelomeric regions, which are known to be prone to homoeologous recombination. The same procedure for phylogenetic analysis was applied to the inversions, but no SVs in intragenic regions or chromosomal segments were excluded. The phylogenetic analysis was initially performed using SNPs to test the analysis pipeline. SNPs were converted to binary presence/absence data, and the same workflow used for the collective SVs dataset was applied. The software chose the BIC model GTR2 + FO + ASC + G4. Phylogenetic analyses were performed separately for the A and C subgenomes based on alignments to the D10 reference. Two accessions of *B. rapa* (*B. rapa* ssp. *rapa* and *B. rapa* ssp. *broccolieto*) [71], and two accessions of *B. oleracea* (Genebank/cultivar IDs HRIGRU007343 and HRIGRU007338) [35] as well as genome assemblies of *B. rapa* Z1 (yellow sarson) and *B. oleracea* HDEM (broccoli) [72], were used to represent diploid progenitors. The reliability of the ML trees was estimated using the ultrafast bootstrap approach (UFboot) with 1000 replicates, and the consensus ML trees including bootstrap values were visualized using the online tool Interactive Tree of Life (iTOL) v6.8 (https://itol.embl.de). Finally, to assess genome-wide patterns of SV differentiation, principal component analysis (PCA) was performed using the complete binary matrix of merged SV events, separately for collective SVs and inversions, using Plink v1.90b6.21 [95], and visualized with the plotly package v4.10.1 in R [92].

### Characterization of the candidate loci under selection

To identify potential genomic regions under selection, the pairwise fixation index (*F*_ST_; Weir and Cockerham *F*_ST_ estimates) [96] was calculated for pairwise comparisons of swede vs. non-swede, East Asian OSR vs. all other OSR, and WOSR vs. SOSR accessions using the VCFtools suite [97] across non-overlapping 50-kbp windows. The analysis was performed separately for collective SVs and inversions. *F*_ST_ values were standardized and transformed into *z*-scores in R [92] using the formula: Z = (*x* − *μ*)/*σ*, where *x* is the *F*_ST_ value for each bin, *μ* is the mean of *F*_ST_ values, and *σ* is the standard deviation of *F*_ST_ values. Genomic regions under selection were identified using a one-tailed *Z*-test with a significance level of *α* = 0.05, corresponding to *Z* = 1.645. Using the intersect function of BEDTools, the results were intersected with the gff file of the D10 reference genome to find genes located in regions under selective signatures, and then merged with annotations from Araport11 using R [92]. For each pairwise comparison, the genetic loci that have *z* values > 0 were used to compare the average *z* values among subgenomes using one-tail Student’s test with a significance level of *α* = 0.01. The GO enrichment analysis was performed using goseq package [98] in R, with a significance level of *α* = 0.05.

### Detecting large duplicated or deleted genomic segments and determining pangenome-wide gene PAV

To detect genomic rearrangements, aligned reads were used to calculate coverage across chromosomes using the bamtobed and genomecov functions from BEDTools, which served as input for a modified deletion-duplication pipeline based on Stein et al. [68]. Briefly, outlier regions with coverage above 150 were discarded, and segments ≥ 10 kbp with coverage deviating by at least one standard deviation above or below the mean were identified as duplications and deletions, respectively. Using the intersect function from BEDTools, the results were compared with the gff file of the D10 reference genome to detect genes affected by large chromosomal segments that were either deleted or duplicated.

To identify genes whose presence in these deleted or duplicated regions correlated with ecotype diversification among WOSR, SOSR, East Asian OSR, and swede accessions, the following criteria were applied: ecotype of interest ≥ 70%, other ecotypes ≤ 30%. The filtering was performed in R [92]. The results were then merged with Araport11 gene functional annotations using R [92]. East Asian OSR accessions were not included as “other ecotypes,” as some accessions from this group clustered closely with WOSR and SOSR accessions in principal component and phylogenetic analyses.

### Annotation and analysis of TEs

To annotate the potential overlap of TEs with insertions and inversions and also explore whether transposable elements might be drivers of SV formation, the *Brassica napus* transposable element library, first described by Chalhoub et al. [4], was downloaded from the supplementary files provided by Rousseau-Gueutin et al. [44] and used to create a local database with BLASTn [88]. Then, in two separate procedures for insertions and inversions, their sequence and the sequences 100 bp upstream and downstream of their putative breakpoints were blasted against the local database (parameters: -evalue 1e-4 -perc_identity 99 -ungapped -max_target_seqs 1 -max_hsps 1).

### Characterization of sequence motifs in SV breakpoints

For characterizing the SV-breakpoints of merged SVs across the panel, sequences 100 bp upstream and downstream of each putative SV-breakpoint were extracted. The analysis was performed using the motif finder MEME v5.4.1 [99] as described by Samans et al. [5]. For the collective SVs, merged SVs were grouped into seven different size ranges: 30–500 bp, 501–1000 bp, 1001–2000 bp, 2001–3000 bp, 3001–10,000 bp, 100,001–30,000 bp, 30,001–100,000 bp to account for the possibility of different SV-origin mechanisms.

### Saturation analysis

We performed a saturation curve analysis to assess the extent to which our set of 94 accessions captures the pangenomic diversity of *B. napus*. Using 88,288 genes that overlapped SNPs, identified by intersecting gene models with the pangenome-wide SNP data across 94 accessions, we randomly sampled increasing numbers of genomes at a maximum value of 94, iterating the process 1000 times.

### Gene flow analysis

To infer patterns of gene flow among seven *B. napus* groups (Table S1), we employed TreeMix v1.13 [100]. A total of 861,307 SVs from collective SVs were processed to construct the input file. TreeMix was run with migration edges ranging from 0 to 6 and WOSR was set as the root (parameters: -m (migration edge number) -k 1000 -bootstrap 1000 -noss -root WOSR). We used parameter -k 1000, which constructs the tree using blocks of 1000 SVs to account for linkage disequilibrium. We built trees using 0 to 6 migration edges and generated a residual heatmap for each migration edge to identify groups that were not well modeled after adding each migration edge. The optimal number of migration edges was determined using the OptM R package [101]. This analysis identified four migration edges (*m* = 4) as the most optimal number.

## Supplementary Information

Additional file 1. Supplementary Tables. Supplementary Table 1. List of 94 ecogeographically diverse rapeseedaccessions selected from the ERANET-ASSYST diversity set. Supplementary Table 2. Summary of long-read DNA resequencing data by Oxford Nanopore Technology for 94 *B. napus* accessions. Supplementary Table 3. Genome assembly statistics for 94 *B. napus* accessions. Supplementary Table 4. Number of removed SVs in each chromosome in the process of filtering-out SVs that are likely to overlap with a deleted chromosome segment. Supplementary Table 5. Number of removed SVs in the process of filtering-out SVs that are likely to overlap a deleted chromosome segment. Supplementary Table 6. Number of SVs called for each *B. napus* accession by aligning long-reads to Darmor-bzh v10 reference genome. Supplementary Table 7. Number of total SVs called for each subgenome of *B. napus* accession by aligning long reads to Darmor-bzh v10 reference genome. Supplementary Table 8. Overview of pangenome-wide distribution and frequency of collective SVs in *B. napus*. Supplementary Table 9. Overview of genes intersecting pangenome-wide collective SVs in *B. napus*. Supplementary Table 10. Overview of putative flowering-time-related genes intersecting pangenome-wide collective SVs in *B. napus*.  Supplementary Table11. Overview of putative R genes intersecting pangenome-wide collective SVs in *B. napus*. Supplementary Table 12. Number of inversions called for each *B. napus* accession by aligning reference-guided genome assemblies to Darmor-bzh v10 reference genome. Supplementary Table 13. Overview of pangenome-wide distribution and frequency of inversions in *B. napus*. Supplementary Table 14. Overview of distribution and frequency of over-represented or under-represented inversion events across *B. napus* pangenome for different ecotype groups. Supplementary Table 15. Fisher’s test analysis for distribution and statistical association by ecotype groups for over-represented or under-represented inversion events across *B. napus* pangenome. Supplementary Table 16. Overview of collective SV hotspots across *B. napus* pangenome. Supplementary Table 17. Overview of inversion hotspots across *B. napus* pangenome. Supplementary Table 18. *F*_ST_ Values for pairwise comparison of swede versus non-swede accessions using pangenome-wide collective SVs in *B. napus*. Supplementary Table 19. *F*_ST_ Values for pairwise comparison of East Asian OSR versus all other OSR accessions using pangenome-wide collective SVs. Supplementary Table 20. *F*_ST_ Values for pairwise comparison of WOSR versus SOSR accessions using pangenome-wide collective SVs. Supplementary Table 21. Overview of putative genes intersecting genomic regions under selection for pairwise comparison of swede versus non-swede accessions using pangenome-wide collective SVs. Supplementary Table 22. Overview of putative genes intersecting genomic regions under selection for pairwise comparison of East Asian OSR versus all other OSR accessions using pangenome-wide collective SVs. Supplementary Table 23. Overview of putative genes intersecting genomic regions under selection for pairwise comparison of WOSR versus SOSR accessions using pangenome-wide collective SVs. Supplementary Table 24. GO enrichment analysis for genes overlapping with genomic regions under selection for pairwise comparison of swede versus non-swede accessions for pangenome-wide collective SVs. Supplementary Table 25. GO enrichment analysis for genes overlapping with genomic regions under selection for pairwise comparison of East Asian OSR versus all other OSR accessions for pangenome-wide collective SVs. Supplementary Table 26. GO enrichment analysis for genes overlapping with genomic regions under selection for pairwise comparison of WOSR versus SOSR accessions for pangenome-wide collective SVs. Supplementary Table 27. *F*_ST_ Values for pairwise comparison of swede versus non-swede accessions using pangenome-wide inversions in *B. napus*. Supplementary Table 28. *F*_ST_ Values for pairwise comparison of East Asian OSR versus all other OSR accessions using pangenome-wide inversions. Supplementary Table 29. *F*_ST_ Values for pairwise comparison of WOSR versus SOSR accessions using pangenome-wide inversions. Supplementary Table 30. Overview of putative genes intersecting genomic regions under selection for pairwise comparison of swede versus non-swede accessions using pangenome-wide inversions. Supplementary Table 31. Overview of putative genes intersecting genomic regions under selection for pairwise comparison of East Asian OSR versus all other OSR accessions using pangenome-wide inversions. Supplementary Table 32. Overview of putative genes intersecting genomic regions under selection for pairwise comparison of WOSR versus SOSR accessions using pangenome-wide inversions. Supplementary Table 33. GO enrichment analysis for genes overlapping with genomic regions under selection for pairwise comparison of swede versus non-swede accessions for pangenome-wide inversions. Supplementary Table 34. GO enrichment analysis for genes overlapping with genomic regions under selection for pairwise comparison of East Asian OSR versus all other OSR accessions for pangenome-wide inversions. Supplementary Table 35. GO enrichment analysis for genes overlapping with genomic regions under selection for pairwise comparison of WOSR versus SOSR accessions for pangenome-wide inversions. Supplementary Table 36. Overlapping regions of pangenome-wide collective SVs and inversions under selection in swede versus non-swede comparison. Supplementary Table 37. Overlapping regions of pangenome-wide collective SVs and inversions under selection in Asian OSR versus all other OSR comparison. Supplementary Table 38. Overlapping regions of pangenome-wide collective SVs and inversions under selection in WOSR versus SOSR comparison. Supplementary Table 39. Overview of pangenome-wide distribution and frequency of gene presence-absence variation in *B. napus*. Supplementary Table 40. Overview of pangenome-wide gene presence-absence that correlate with WOSR, SOSR, East Asian OSR, or swede group of accessions.

Additional file 2: Fig. S1. Phylogenetic tree of A and C subgenomes constructed from pangenome-wide SNPs across 94 homozygous, ecogeographically diverse *B. napus* accessions. Fig. S2. Motifs enriched around putative breakpoints of pangenome-wide insertion and deletion events in diverse accessions of *B. napus*. Fig. S3. Motifs enriched around putative breakpoints of pangenome-wide inversions in diverse accessions of *B. napus*. Fig. S4. Saturation curve analysis of pangenomic diversity in 94 accessions of *B. napus.* 

## Data Availability

All raw data generated in this study including long-read DNA sequence data are available on the NCBI BioProject database under accession number PRJNA1183293 at https://www.ncbi.nlm.nih.gov/sra/PRJNA1183293 [102]. Genome assemblies constructed in this study are available on the European Nucleotide Archive under accession number PRJEB82438 at http://www.ebi.ac.uk/ena/browser/view/PRJEB82438 [103]. The following data are downloaded and used for phylogenetic analysis in this study. Two accessions of *B. rapa* (*B. rapa* ssp. *rapa* and *B. rapa* ssp. *broccolieto*) are available at https://www.ncbi.nlm.nih.gov/bioproject/PRJNA730930 [71, 104] and two accessions of wild *B. oleracea* (Genebank/cultivar ID: HRIGRU007343 and HRIGRU007338) are available at https://www.ncbi.nlm.nih.gov/bioproject/PRJNA1047966 [35]; *B. rapa* accession Z1 and *B. oleracea* accession HDEM are available at http://www.genoscope.cns.fr/plants [72]. No other scripts and software were used other than those mentioned in the Methods section.
