# Supplementary material for: Pangenomic structural variant patterns reflect evolutionary diversification in Brassica napus
Source: Genome Biol. 2025 Nov 10;26:381. doi: 10.1186/s13059-025-03833-x (PMC12599057; doi:10.1186/s13059-025-03833-x)
Supplement: Supplementary file 2 — Additional file 2: Fig. S1. Phylogenetic tree of A and C subgenomes constructed from pangenome-wide SNPs across 94 homozygous, ecogeographically diverse B. napus accessions. Fig. S2. Motifs enriched around putative breakpoints of pangenome-wide insertion and deletion events in diverse accessions of B. napus. Fig. S3. Motifs enriched around putative breakpoints of pangenome-wide inversions in diverse accessions of B. napus. Fig. S4. Saturation curve analysis of pangenomic diversity in 94 accessions of B. napus. [file 13059_2025_3833_MOESM2_ESM.pdf]

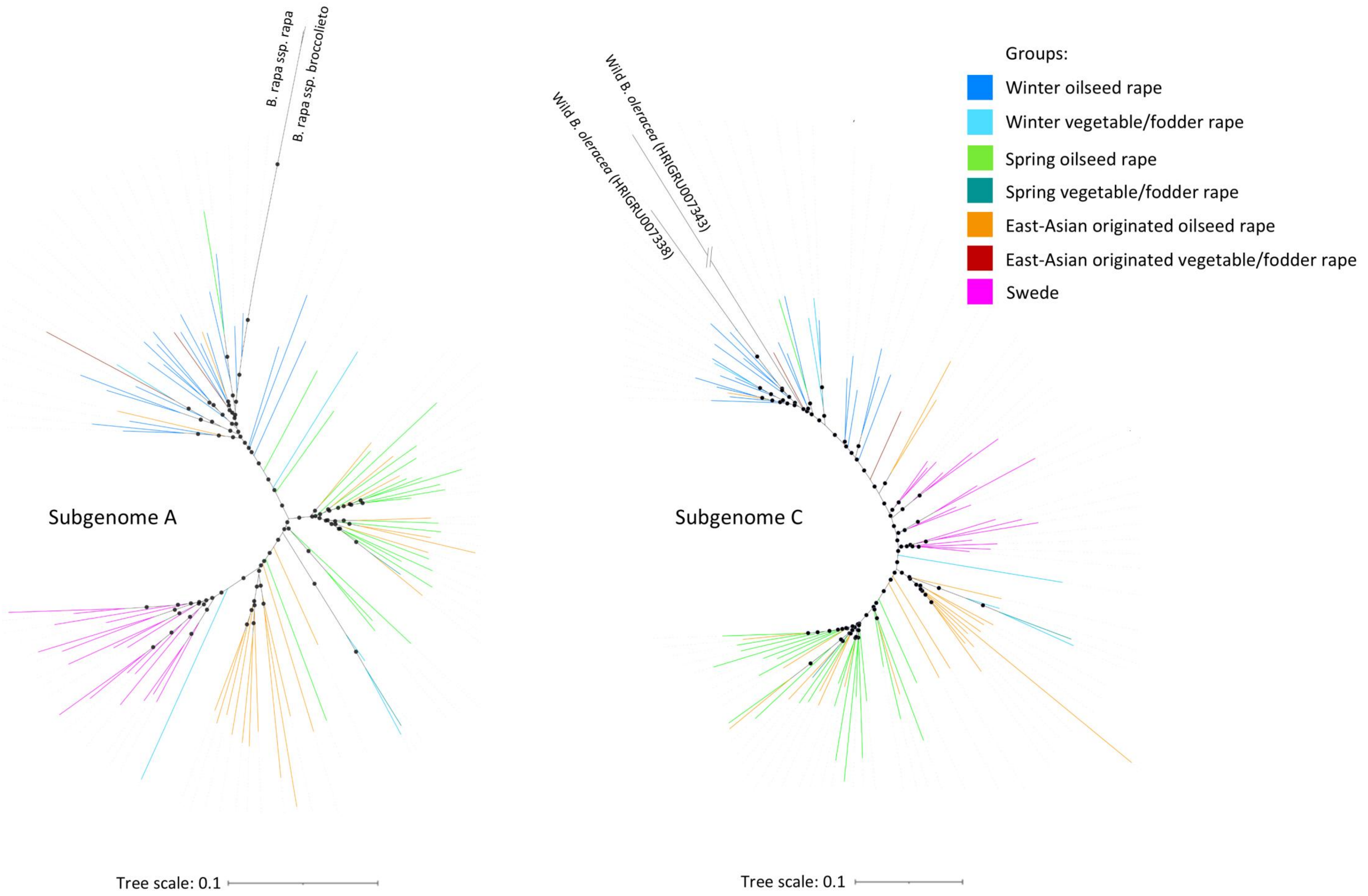

Figure S1: Phylogenetic tree of A and C subgenomes constructed from pangenome-wide SNPs across 94 homozygous, ecogeographically diverse *B. napus* accessions. Two accessions of *B. rapa* (*B. rapa* ssp. *rapa* and *B. rapa* ssp. *broccolieto*) [71] and two accessions of wild *B. oleracea* (Genebank/cultivar ID: HRIGRU007343 and HRIGRU007338) [35] were included in the analysis for the A and C lineages, respectively. Phylogenetic trees are constructed using a maximum likelihood approach. The reliability of the tree was also confirmed by 1000 bootstrap replicates, and clades with bootstrap values of above 50% are indicated by a black dot. Visualization was performed using the Interactive Tree Of Life online tool (https://itol.embl.de).

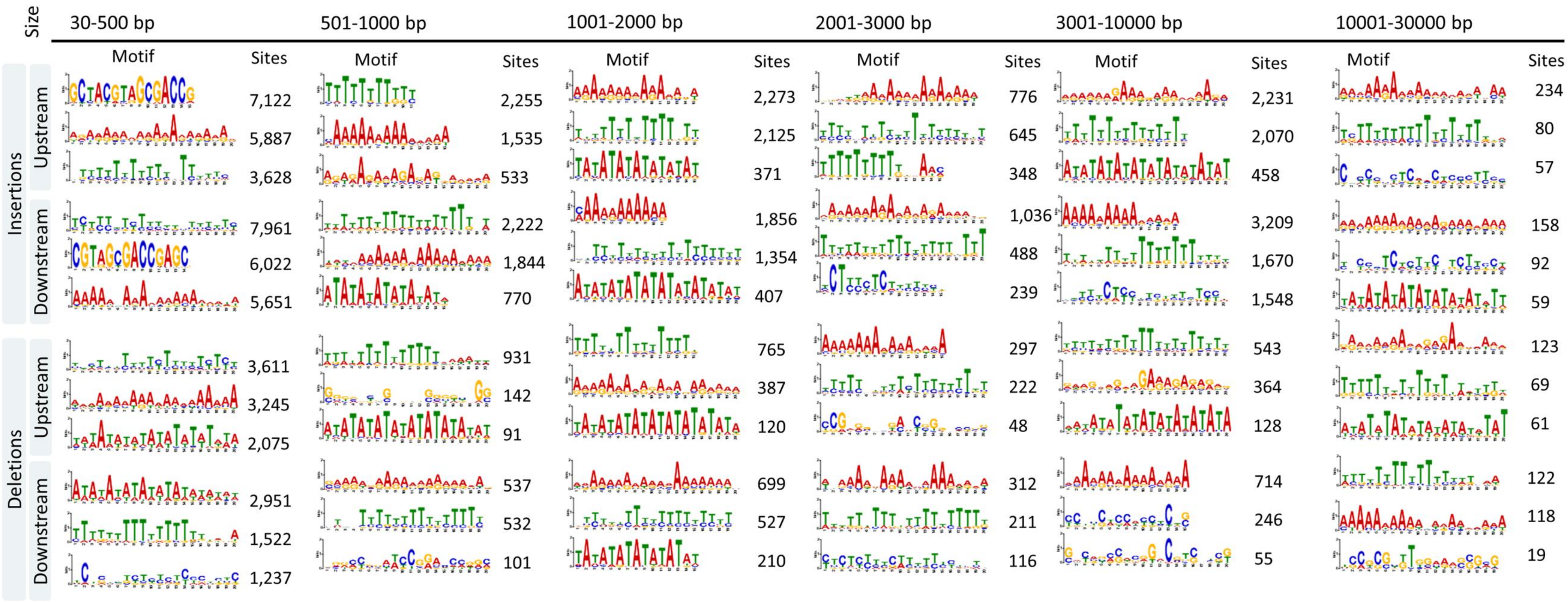

Figure S2: Motifs enriched around putative breakpoints of pangenome-wide insertion and deletion events in diverse accessions of *B. napus*. For each breakpoint, the genomic sequence of 100 bp upstream and downstream was extracted from six different size categories of SVs and analyzed for enriched DNA motifs using MEME software, with accepted e-values <0.05. For each SV size group, the three most abundant motifs (by the number of sites) are shown for both the downstream and upstream regions of the SVs.

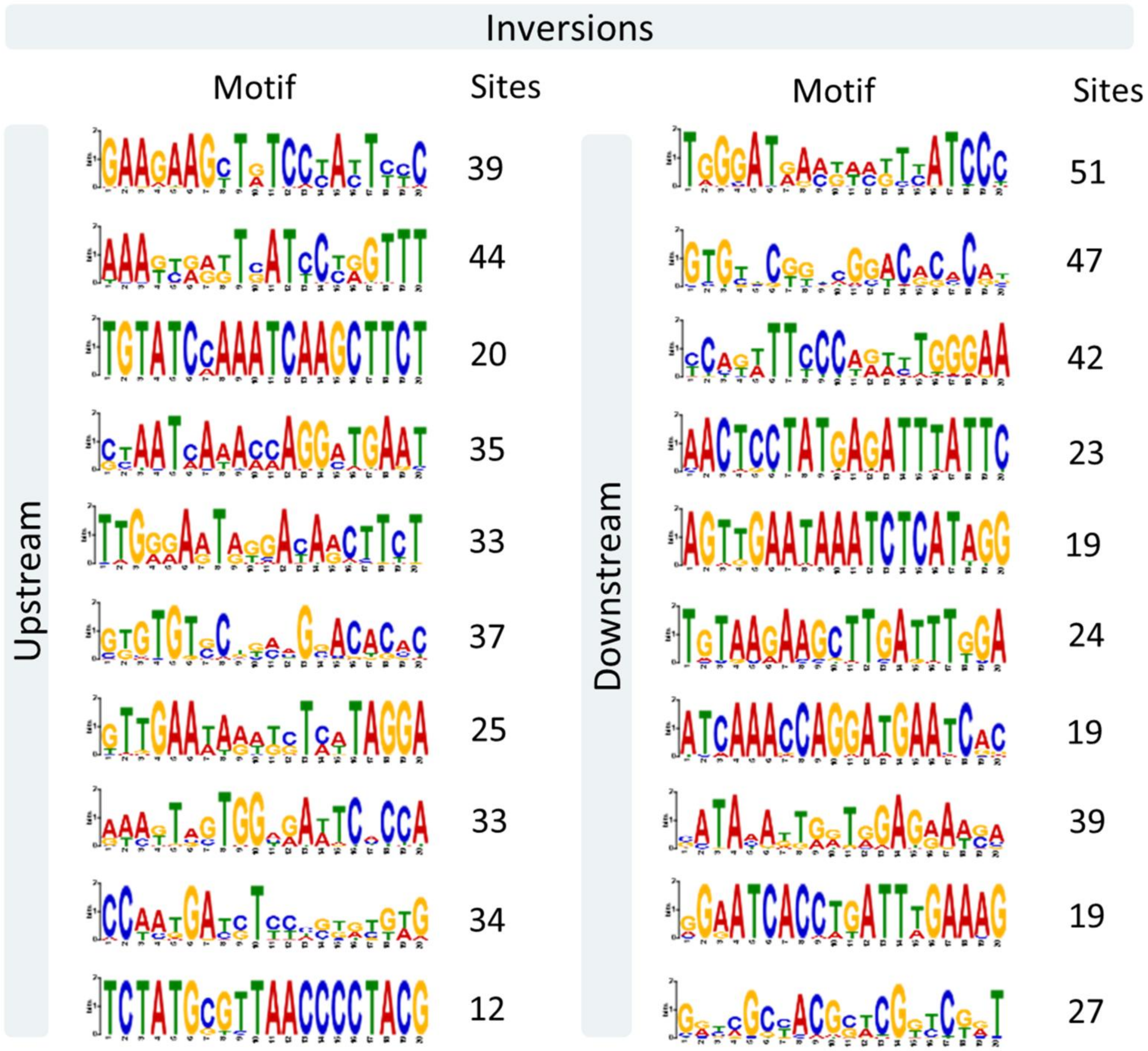

Figure S3: Motifs enriched around putative breakpoints of pangenome-wide inversion events in diverse accessions of *B. napus*. For each breakpoint, the genomic sequence of 100 bp upstream and downstream was extracted and analyzed for enriched DNA motifs using MEME software, with accepted e-values <0.05.

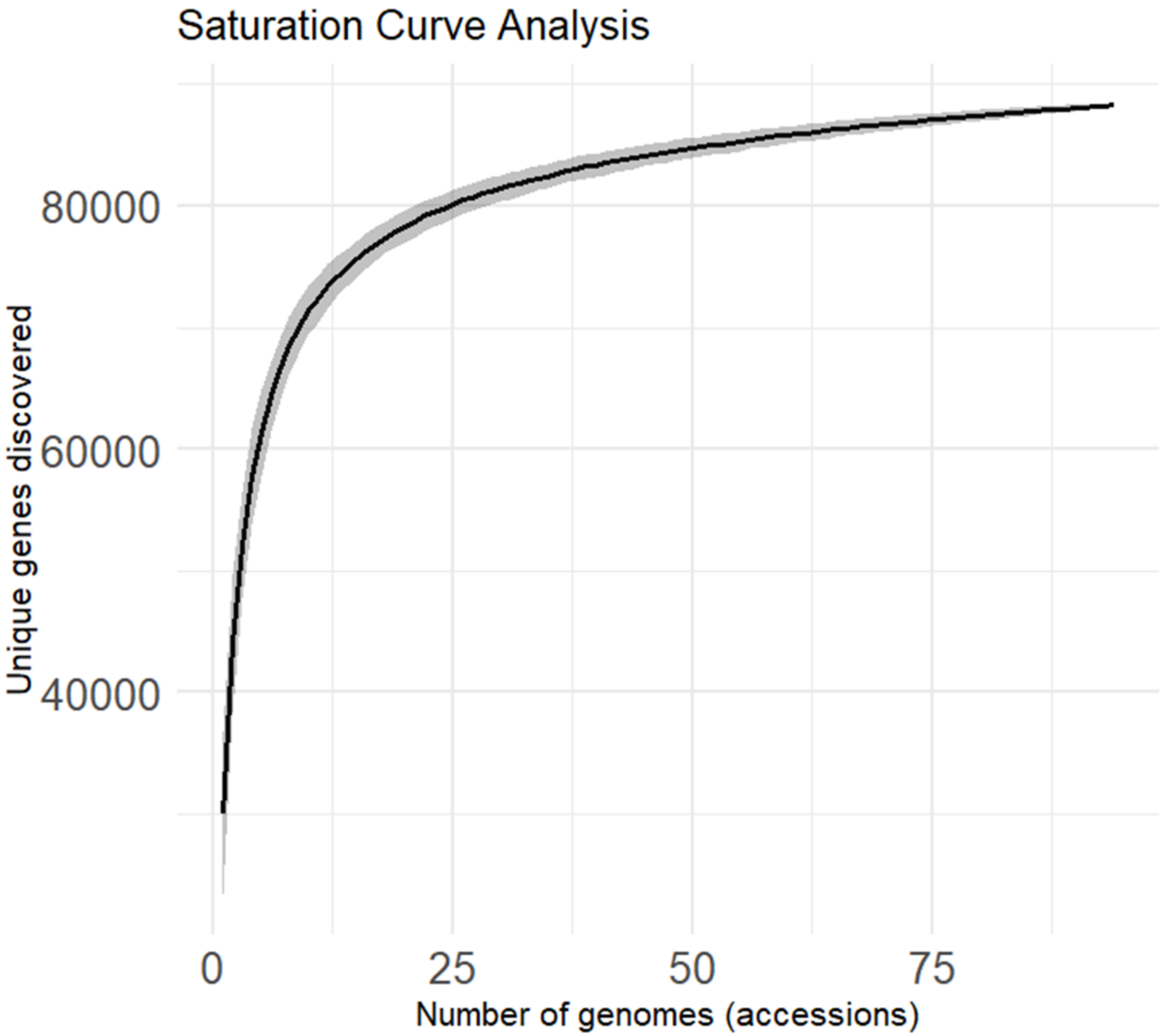

Figure S4: Saturation curve analysis of pangenomic diversity in 94 accessions of *B. napus*. The x-axis shows the number of unique genes overlapping SNPs discovered and the y-axis presents the number of genomes (accessions) included. The analysis was based on 1,000 iterations of random sampling across the 94 accessions.
